# Characterization of the *TCP* Gene Family in *Chrysanthemum nankingense* and the Role of *CnTCP4* in Cold Tolerance

**DOI:** 10.3390/plants11070936

**Published:** 2022-03-30

**Authors:** Chang Tian, Lisheng Zhai, Wenjing Zhu, Xiangyu Qi, Zhongyu Yu, Haibin Wang, Fadi Chen, Likai Wang, Sumei Chen

**Affiliations:** 1 State Key Laboratory of Crop Genetics and Germplasm Enhancement, Key Laboratory of Flower Biology and Germplasm Innovation, Ministry of Agriculture and Rural Affairs, Key Laboratory of Biology of Ornamental Plants in East China, National Forestry and Grassland Administration, College of Horticulture, Nanjing Agricultural University, Nanjing 210095, China; 2015204031@njau.edu.cn (C.T.); 2019204048@njau.edu.cn (L.Z.); 2017104094@njau.edu.cn (W.Z.); 2012204028@njau.edu.cn (X.Q.); 2019204044@njau.edu.cn (Z.Y.); hb@njau.edu.cn (H.W.); chenfd@njau.edu.cn (F.C.); 2 Shenzhen Branch, Guangdong Laboratory for Lingnan Modern Agriculture, Genome Analysis Laboratory of the Ministry of Agriculture, Agricultural Genomics Institute at Shenzhen, Chinese Academy of Agricultural Sciences, Shenzhen 518120, China

**Keywords:** TCP transcription factors, phylogeny analysis, expression profiles, cold tolerance

## Abstract

Plant-specific TCP transcription factors play a key role in plant development and stress responses. *Chrysanthemum nankingense* shows higher cold tolerance than its ornamental polyploid counterpart. However, whether the *TCP* gene family plays a role in conferring cold tolerance upon *C. nankingense* remains unknown. Here, we identified 23 *CnTCP* genes in *C. nankingense*, systematically analyzed their phylogenetic relationships and synteny with TCPs from other species, and evaluated their expression profiles at low temperature. Phylogenetic analysis of the protein sequences suggested that CnTCP proteins fall into two classes and three clades, with a typical bHLH domain. However, differences between *C. nankingense* and Arabidopsis in predicted protein structure and binding sites suggested a unique function of CnTCPs in *C. nankingense*. Furthermore, expression profiles showed that expression of most *CnTCPs* were downregulated under cold conditions, suggesting their importance in plant responses to cold stress. Notably, expression of *miR319* and of its predicted target genes, *CnTCP2*/*4*/*14*, led to fast responses to cold. Overexpression of Arabidopsis *CnTCP4* led to hypersensitivity to cold, suggesting that *CnTCP4* might play a negative role in *C. nankingense* responses to cold stress. Our results provide a foundation for future functional genomic studies on this gene family in chrysanthemum.

## 1. Introduction

The *TCP* gene family is a plant-specific group of transcription factors (TFs) that was first described in 1999 [1,2]. The family name is derived from four proteins, namely, TB1 (teosinte branched 1) in maize [3], CYC (CYCLOIDEA) in Antirrhinum [4], and PCF1 and PCF2 (Proliferating cell factors) in rice [2]. Furthermore, *TCP* genes have been found in various plant species, for instance, model plants such as *Arabidopsis* [1,5,6,7]; crop plants such as soybeans [8], maize [9] and rice [7]; and pluricellular green algae [10,11] such as cosmarium and chara, moss *Physcomitrium patens*, ferns and lycophyte *selaginella*, poplar (*Populus*) [12], and grapevine [13]. Furthermore, members of this gene family play numerous roles in plant development, including in embryonic growth [14], cell cycle regulation [15,16], pollen development [17], germination [14,18], senescence [19], circadian rhythms [20,21], leaf development [12,22,23,24,25], branching [26,27,28], floral organ morphogenesis and flowering [29,30,31,32,33,34], and hormone signaling [8,14,31,35,36,37].

Class II CIN-type *TCPs*, as *miR319* targets, have been widely analyzed [5,19]. *TCPs* regulated by *miR319* reportedly coordinate plant physiological characteristics such leaf growth and senescence [33], shoot apical meristem development [38], flowering time [33], and cold tolerance [39]. TCP proteins harbor a bHLH motif that allows DNA binding and protein–protein interactions [5,6]. Many studies have shown that TCP transcription factors in Arabidopsis, tomato, and rice [5,6,23,40,41,42,43,44,45] exist as homodimers or heterodimers; furthermore, interaction usually occurs among TCP transcription factors of the same class. Additionally, some studies have reported that TCPs are more likely to form heterodimers than homodimers and that the former bind DNA more efficiently than the latter [2,40,41]. For instance, PCFl and PCF2 bind the rice meristematic tissue-specific expressed *PCNA* gene through homodimers or heterodimers, but the heterodimeric interaction between PCFl and PCF2 is considerably stronger than between the homodimers [2]. Consistently, AtTCP11 and AtTCP15 form heterodimers that show greater efficiency in binding DNA and have a different sequence preference than their corresponding homodimer counterparts [41]. Furthermore, TCP proteins might interact with other non-TCP proteins, thereby affecting the expression of downstream genes and regulating the plant development. For example, Chrysanthemum CmTCP20 interacts with CmJAZ1 and downregulates *CmBPE2* expression, which regulates the petal size [46]. AtTCP5/13/17 directly interact with AtFD to activate *AtAP1* expression and promote the photoperiodic flowering response [30]. These results suggest that the specific roles played by TCPs may be partially dependent on the proteins with which they interact.

Cold stress is one of the main abiotic stress conditions that severely reduce crop productivity, quality, and post-harvest longevity [47]. Cold stress induces transcriptional, post-transcriptional, and post-translational regulation of gene expression [39,48,49,50]. Evidence has shown that the inducer of the *C-repeat binding factor* (*CBF*) *expression 1* (*ICE1*), *CBF*/*DREB1* transcriptional cascade, and *CBF*-independent regulons are involved in transcriptional regulation during cold acclimation [50,51]. Furthermore, *ICE1* is regulated by an ubiquitin-mediated mechanism of translational control upon cold induction [52]; moreover, pre-mRNA splicing, mRNA export, and small RNA-directed mRNA degradation play important roles in cold stress responses by post-transcriptional regulatory mechanisms [39,53,54,55,56].

In addition to a great worldwide popularity as cut flowers and pot plants, chrysanthemums are highly appreciated as health foods and anti-inflammatory herbs of high commercial value in traditional Chinese medicine. During chrysanthemum growth, extremely low temperatures in early spring and winter, unusual freezing temperatures during late spring, and sudden frost during the fall often lead to growth arrest and flower bud or inflorescence inhibition, ultimately causing substantial yield and economic losses [48,57]. Therefore, improving cold tolerance of chrysanthemum is an important goal. The diploid species *Chrysanthemum nankingense* is closely related to the high market-value ornamental species, *C. morifolium*. Due to its simple diploid nature, *C. nankingense* has been selected as a convenient Asteraceae genomic model for rapid and effective exploration of gene function [22,57,58,59,60,61]. Additionally, *C. nankingense* shows greater cold tolerance than its ornamental polyploid counterpart [57,62]. Furthermore, a few *CnTCPs* found in *C. nankingense* are reportedly related to the plant response to low temperature [57]. However, to date, no attempt has been made to systematically describe the *CnTCP*-gene family members harbored by the *C. nankingense* genome. Therefore, this study was designed to systematically analyze and determine the phylogenetic relationships and synteny of *CnTCPs* present in *C. nankingense* with TCPs from other species. Here, we aimed to document the transcriptional behavior of *CnTCPs* under low temperature. Ectopic overexpression of *CnTCP4* in Arabidopsis led to reduced cold tolerance, suggesting that *CnTCP4* plays a negative role in the response of *C. nankingense* to cold.

## 2. Results

### 2.1. Classification and Phylogenetic Analysis of TCP Proteins

**Figure 1:**
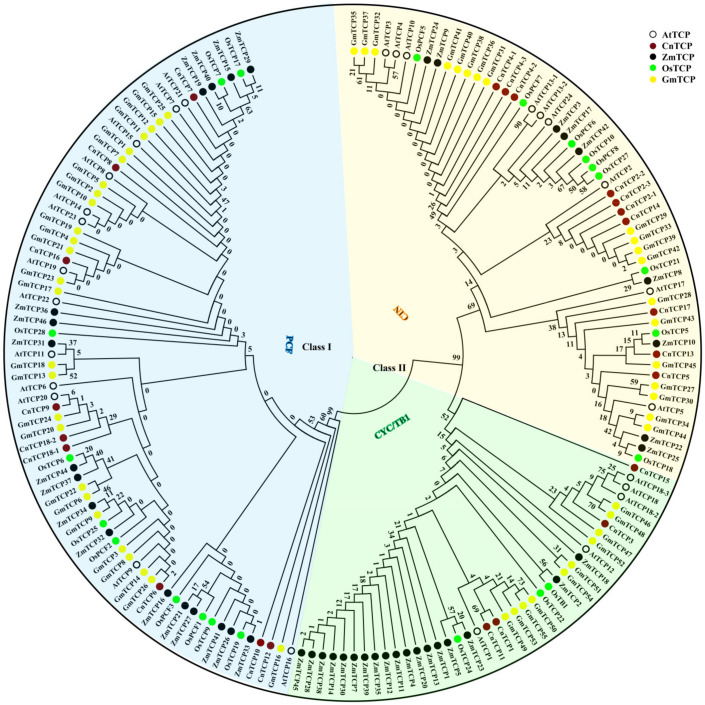
Phylogenetic relationships of CnTCP proteins. The phylogenetic tree was generated using the MEGA7.0 program; the bHLH domain amino acid sequences of TCP proteins from *Glycine max* (Gm), *Oryza sativa* (Os), *Zea mays* (Zm), *Chrysanthemum nankingense* (Cn), and *Arabidopsis thaliana* (At) were used.

A total of 171 TCP proteins were selected for systematic analysis of evolutionary relationships, including 55 from *Glycine max* [8], 22 from *Oryza sativa* [5], 44 from *Zea mays* [9], 23 from *C. nankingense*, and 27 from *Arabidopsis thaliana* [5] (Figure 1). The 171 TCPs were classified into two main classes, i.e., class I and class II, each with three clades. The boundaries of these major clades illustrate the phylogenetic positions of several canonical TCP proteins, such as class I PCF proteins OsPCF1 and OsPCF2, CYC/TB1-like class II protein ZmTB1, and CIN-like class II protein AtTCP2-4. Furthermore, we found that TCP proteins from the three dicot plants used here, namely, Arabidopsis, *G. max*, and *C. nankingense*, seemingly clustered separately from those of the two monocots studied, namely, *O. sativa* and *Z. mays* (Figure 1). Furthermore, TCPs from *G. max* and *C. nankingense* TCPs showed a closer phylogenetic relationship among them than those from maize and rice did.

**Figure 2:**
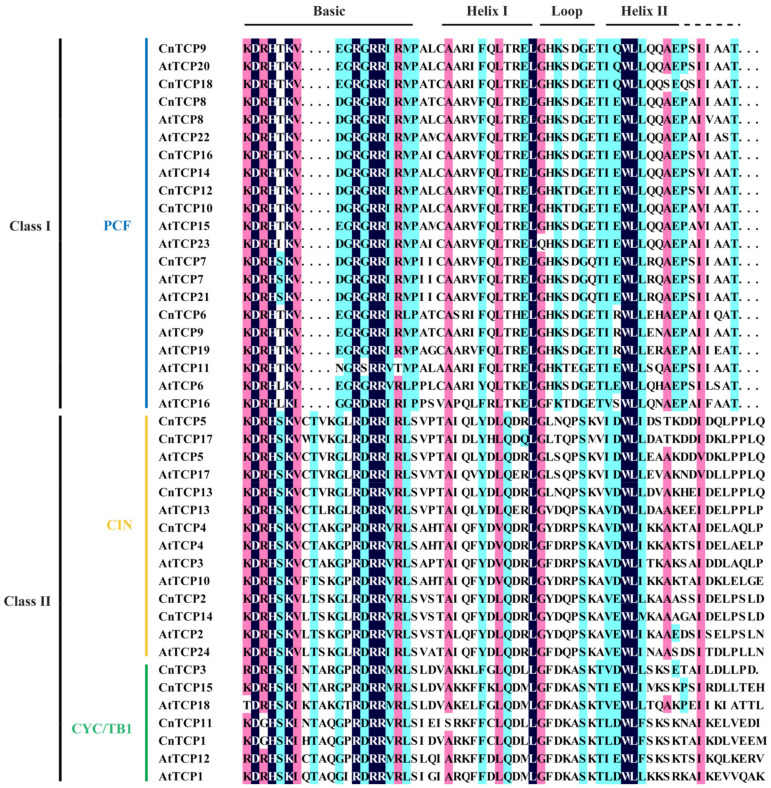
Amino acid alignment among TCP proteins. Amino acids alignment using the bHLH domain amino acid sequences of TCP proteins from *Chrysanthemum nankingense* and *Arabidopsis thaliana*. The blue line shows PCF subfamily in class I; the yellow line shows CIN subfamily in class II; the green line shows CYC/TB1 subfamily in class II.

All CnTCP proteins showed the basic helix loop helix (bHLH) domain (Figure 2). The distance matrix and homology matrix of the full-length and bHLH CnTCP protein sequences from five plant species are shown in Supplementary Tables S2 and S3. High homologies in full length and bHLH domain were noted among proteins (Figure 1). The conserved bHLH domain has a 59-amino acid bHLH structure that allows DNA binding and protein–protein interactions [1,2]. The most significant difference between the two protein classes is the absence of four amino acids in the basic domain of proteins in class I, compared with that of proteins in class II. Additional diagnostic residues for each class were found in the helices and the loop of the TCP bHLH domain (Figure 2). Importantly, our phylogenetic analysis and bHLH domain architecture support the classification of the *C. nankingense* and Arabidopsis *TCP* genes family. Our phylogenetic tree and bHLH domain analyses (Figure 1 and Figure 2) showed that CnTCP14 and AtTCP2 clustered together into the same group and were classified into Class II CIN. In turn, CnTCP3 and CnTCP15 clustered with AtTCP18 and were classified into Class II CYC/TB1; meanwhile, CnTCP10 and CnTCP12 clustered with AtTCP16 and were classified into Class I PCF. Lastly, CnTCP18 clustered with AtTCP20 and was classified into Class I PCF.

Compared with that of Arabidopsis, CnTCP proteins number occupies a lower proportion in the PCF subfamily, a higher proportion in the CIN subfamily, and a similar proportion in the CYC/TB1 subfamily. Specifically, among the five species studied herein, members in Class I PCF accounted for the largest proportion, with 47% in *G. max* (26/55), 39% in *Z. mays* (17/44), 48% in Arabidopsis (13/27), 45% in *O. sativa* (10/22), and 39% in *C. nankingense* (9/23); meanwhile, CYC/TB1 subfamily members accounted for 18% (10/55), 41% (4/22), 19% (5/26), 14% (4/21), and 17% (5/25), respectively. The proportion of CIN members in the five species ranged between 20% and 43% (Figure 1), among which *C. nankingense* and *Z. mays* showed the highest and the lowest proportions, respectively.

Furthermore, we constructed an unrooted phylogenetic tree from alignments of the protein sequences of the bHLH domain from *C. nankingense*, *Helianthus annuus*, and Arabidopsis (Supplementary Figure S1). The Hidden Markov Model (HMM) profile of the TCP domain (PF03634) was used to identify the TCP proteins from *H. annuus*, one of the sequenced Asteraceae species [61,63]. A total of 29 TCP family proteins were identified in the sunflower genome, of which 14 clustered in the CYC/TB1 clades of class II, 9 in the CIN clades of class II, and 6 in the PCF clades of class I (Supplementary Figure S1).

### 2.2. Structural Analysis and Protein–Protein Interactions of CnTCP Genes

**Figure 3:**
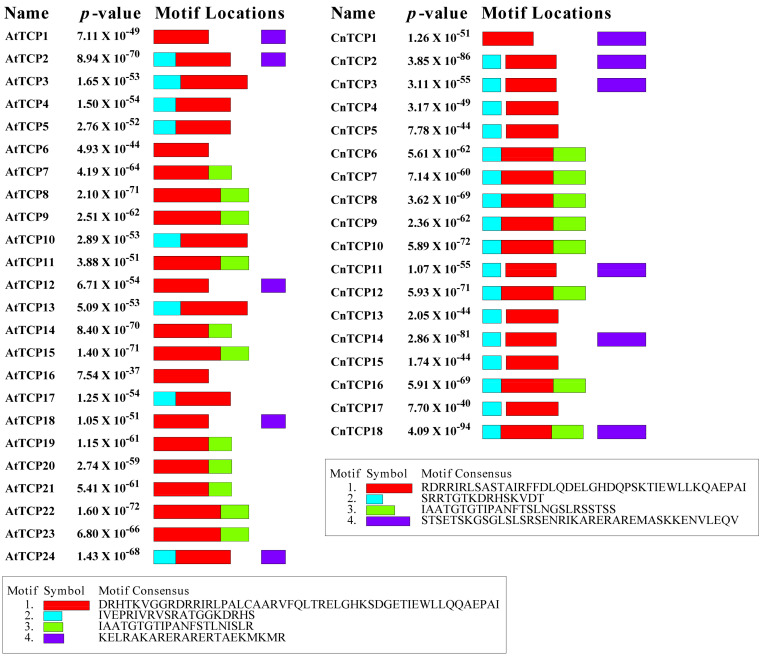
Predicted protein schematic structures of TCPs. The protein structure based on the TCP proteins from *Arabidopsis thaliana* (left) and *Chrysanthemum nankingense* (right); other additional domains are as identified by SMART. The bottom box indicates the motif number, symbol, and the motif consensus sequence of the corresponding domain. Actual motif length and order are presented for each protein.

**Figure 4:**
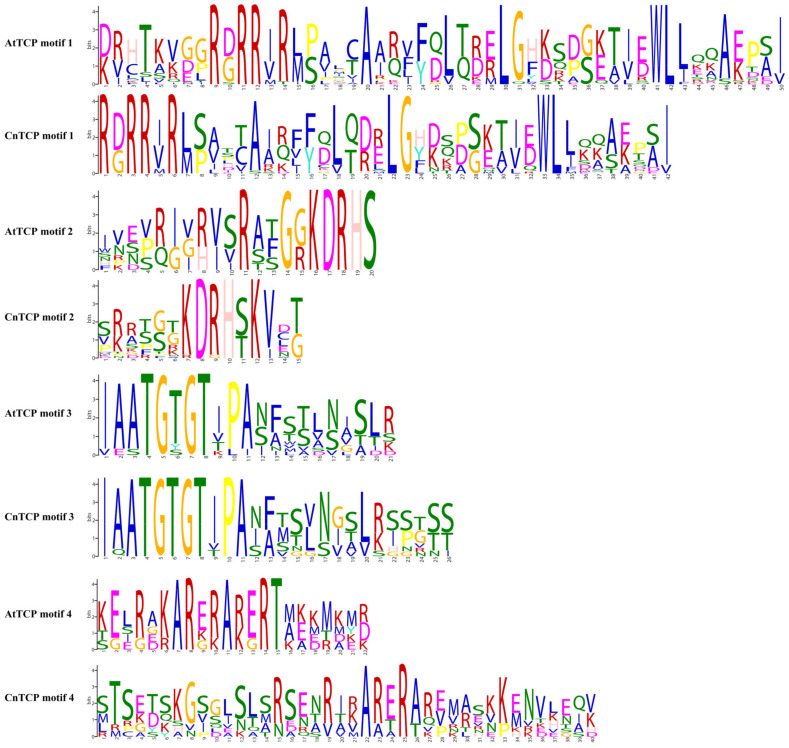
Sequence LOGOs for each motif of TCP domains using the MEME algorithm. The motif consensus of the predicted domain in CnTCP and AtTCP proteins. The height of a single letter in the stack indicates the probability of that letter in that position multiplied by the total information content of the stack.

Family pair-wise analysis of the full-length CnTCP protein sequences indicated that the identities ranged from 9.40% to 68.10% in non-homologous gene pairs (Table 1). Relatively high identities were observed between CnTCP10 and CnTCP12 (68.10%), CnTCP2 and CnTCP14 (approximately 57.00%), CnTCP1 and CnTCP11 (52.30%), CnTCP5 and CnTCP17 (52.10%), and among CnTCP16, CnTCP10 and CnTCP12 (approximately 50.00%). Additionally, TCP-protein family pair-wise analysis among the sequences of the bHLH domain showed much higher homologies, e.g., 98.20% between CnTCP10 and CnTCP12 (Supplementary Table S4). Such a high degree of homology suggests that these two proteins may have similar structures and function. The structure of each AtTCP and CnTCP proteins are shown in Figure 3. The MEME motif search tool was used to identify the conserved motifs of TCPs domains in *C. nankingense* and Arabidopsis. Among the four distinct motifs identified, motif 1 was located in the bHLH domain (Figure 4), whereas motifs 2 and 3 were located at the upstream and downstream parts of motif 1, respectively. The amino acids in the front and back of motifs 2 and 3 were relatively conserved and located in the bHLH domain, while the other parts showed larger variation (Figure 3 and Figure 4). The highly divergent, fast-evolving sequences outside the bHLH domain are essential for protein functional specificity. Outside the bHLH domain, an 18–20 amino acid residue, arginine-rich motif is also conserved in Arabidopsis and is specific to a subset of class II proteins (the R domain, motif 4) (Figure 3 and Figure 4); this motif was predicted to form a coil that may mediate protein–protein interactions [5]. For instance, AtAP1 interacts with AtTCP24 but not with the closely related AtTCP3/5/13/17 proteins that also harbor almost identical TCP domains [64]. The R domain in Arabidopsis differed from its counterpart in *C. nankingense* in the number of amino acid residues and, consequently, related characteristics (Figure 3 and Figure 4). Consistent with phylogenetic analysis and domain architecture, CnTCP3 and CnTCP14 harboring the R domain clustered together into the Class II subfamily (Figure 1 and Figure 2). However, CnTCP18 harboring a predicted R domain clustered into Class I PCFs but lacked four amino acids in the basic bHLH domain. The differences in protein structure and motif prediction between *C. nankingense* and Arabidopsis might entail a unique function of CnTCP in *C. nankingense*.

Untranslated regions (UTRs) and introns present in genes may play regulatory roles, such as modulating mRNA stability or localization and translational efficiency [5,65,66,67]. Furthermore, introns can increase transcript levels by affecting the rate of transcription, nuclear export, and transcript stability, and by increasing the efficiency of mRNA translation [68]. Some *AtTCP* genes such as *AtTCP1*/*8/12*/*13/18* have introns, while *AtTCP12* and *AtTCP24* have a long 3′ UTR and 5′ UTR. Although the annotation file of *C. nankingense* is not complete, several *CnTCPs*, such as *CnTCP14/17/18*, harbor long introns (Supplementary Figure S2).

Protein–protein interactions are the basis on which cellular structure and function are built, and interacting partners directly affect biological functions of each other. Here, the STRING online database (accessed on 28 October 2021, https://string-db.org/) and Cytoscape software were used to analyze the interactions between Arabidopsis TCPs and other proteins. A total of 123 proteins were filtered into a PPI network complex containing 835 edges (Supplementary Figure S3A). PPI networks revealed that cold stratification induced TCPs’ interactions with proteins GID1A and GID1B (Supplementary Figure S3A). TCP proteins were interacted with some circadian clock-related genes, such as TCP7/20/21/22 with TOC1, TCP18 and FT, and TCP7/11/22 and CCA1 (Supplementary Figure S3A). In this study, top one protein–protein interaction networks with the highest clustering score were established via MCODE analysis (Supplementary Figure S3B); furthermore, the top module comprised 19 proteins, including SPL/NZZ and most AtTCP proteins (Supplementary Figure S3B), which showed a complex interactive network of the TCP family.

### 2.3. Expression Profiling of CnTCP Genes in Response to Cold Stress

Some *TCP* genes are involved in plant abiotic stress responses, such as in cold [39], salt [8], and drought stress [8,9]. At the molecular level, such changes induced by adverse conditions are mainly mediated by transcription factors binding to specific recognition sequences upstream from the specific stress response genes (*cis*-elements) for transcriptional regulation. In this study, to further elucidate the possible regulatory mechanisms underlying the expression of *CnTCP* genes during abiotic/biotic stress responses, the corresponding promoter sequences were analyzed using the PlantCARE online database to search *cis*-elements in the promoter region within 3000 bp upstream from the initiation codons of *TCPs*. At least 60 *cis*-regulatory elements were identified in *CnTCP* and *AtTCP* promoters (Supplementary Table S5). The upstream regions of most *CnTCP* and *AtTCP* genes contained at least one phytohormone-related element, such as abscisic acid-responsive element (ABRE), ethylene-responsive element (ERE), gibberellin-responsive element (P-box), MeJA-responsive element (CGTCA-motif and TGACG-motif), and salicylic acid-responsive element (TCA element) (Supplementary Table S6). Our results showed the same trend previously observed in five legume species [8]. Furthermore, stress-related elements were found in the promoters of *CnTCPs* and *AtTCPs*, such as low-temperature responsive (LTR) elements, drought-inducibility (MBS), and defense and stress responsiveness TC-rich repeats (Supplementary Table S6). These results indicate that *TCP* genes might play critical roles in plant responses to a range of abiotic stress conditions [9,69]. Furthermore, most *CnTCPs* and *AtTCPs* promoters seemingly harbor the predicted binding sites of transcription factors such as AP2, ERF, bHLH, MYB, WRKY, and TCP (Table 2). There are several additional binding sites that contribute to cold stress mitigation, such as the ABA-responsive elements (ABRE), the G-box, and the MYC binding sites (Supplementary Table S6).

**Figure 5:**
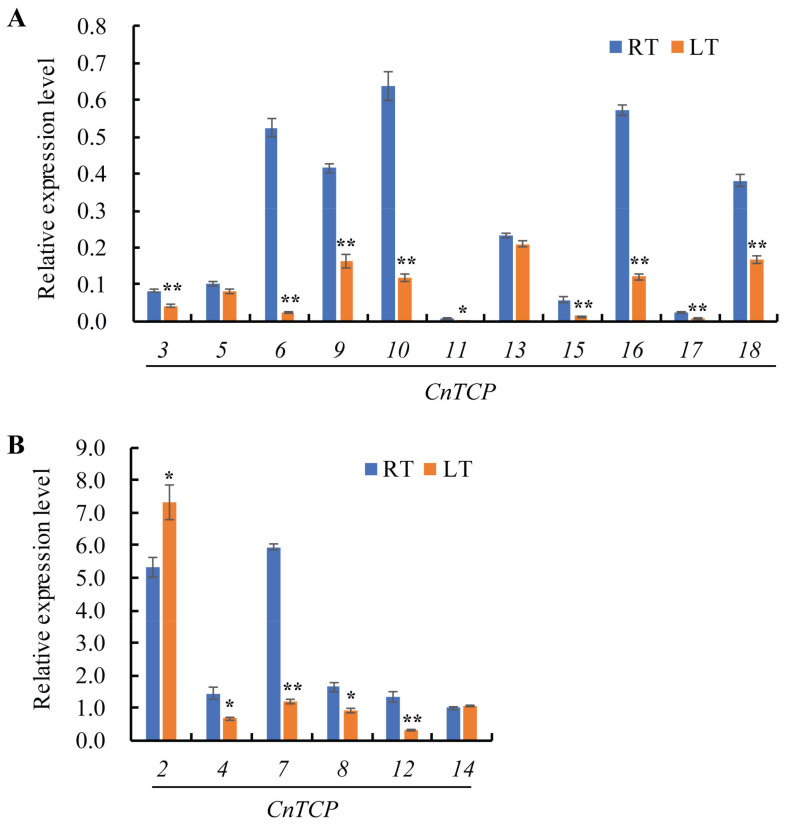
qRT-PCR analysis of *CnTCP* gene expression under cold stress. (**A**,**B**) Expression pattern of the *CnTCP* genes after a 7-day low temperature (4 °C, LT) treatment. The expression level of *CnTCP14* at 22 °C room temperature (RT) was set at 1.0 due to its medium expression level, which could better show the expression pattern of *TCP* family genes. The *EF-1α* gene was used as an internal control. For readability, *CnTCPs* with lower expression levels are included in panel A, while the ones with higher expression levels are included in panel B. One and two asterisks represent significant difference at *p* < 0.05 and *p* < 0.01, respectively, according to the paired-samples *t*-test.

Gene expression patterns provide important information for gene function analysis. Downregulation of the expression of two *TCP* genes in rice, *OsPCF5*, and *OsPCF8* results in enhanced cold tolerance [54]. We tested the transcriptional behavior of *CnTCPs* after a 7 d cold stress in *C. nankingense*. As shown in Figure 5, the expression level of *CnTCP14* under 22 °C room temperature (RT) was comparable to that under 4 °C low temperature (LT); furthermore, when the expression level of *CnTCP14* under RT was used as the normalized control, most of the *TCP* genes’ expression levels were downregulated under 4 °C low temperature, including *CnTCP3*/*4*/*6*/*7*/*8*/*9*/*10*/*11*/*12*/*15*/*16*/*17*/*18* (i.e., LT treatment). The results have shown that *CnTCP5*/*13*/*14* have no expression changed after cold tolerance, while *CnTCP2* upregulated (Figure 5). Our phylogenetic tree and bHLH domain analyses (Figure 1 and Figure 2) showed that CnTCP2/4/5/13/14/17 clustered together into the Class II CIN group. Although the CnTCP4/5/13/17 proteins have the same protein structure, as well as CnTCP2/14 (Figure 3), the bHLH motif sequence differences (Figure 2) may lead to differences in their gene functions.

**Figure 6:**
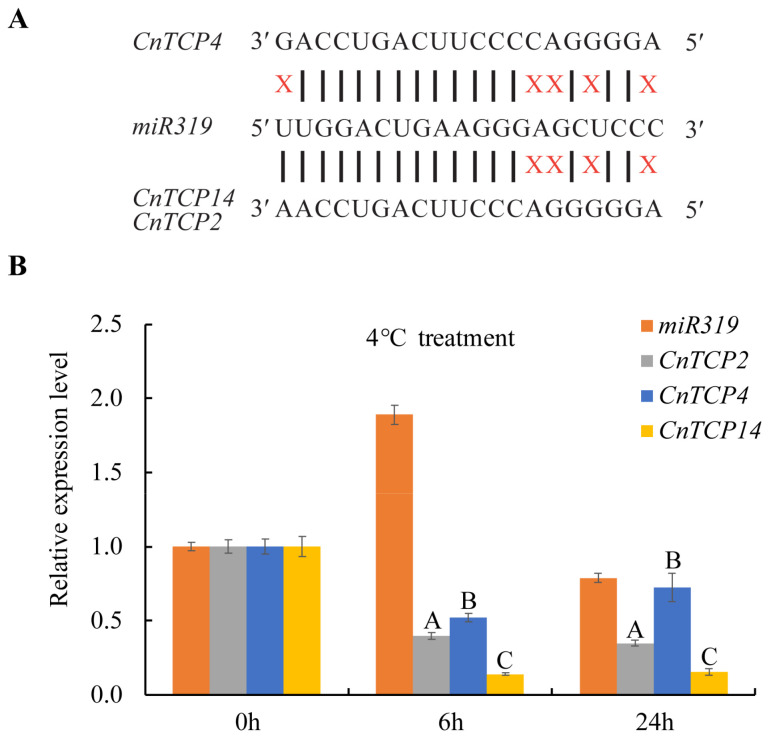
Mature *miR319* and predicted target *CnTCP* expression in response to cold stress. (**A**) Predicted *CnTCP* transcript targets of *miR319*. (**B**) Expression levels of mature *miR319* and its predicted targets *CnTCP2/4/14* in two-month-old *Chrysanthemum nankingense* plants treated at 4 °C for 0, 6, and 24 h. *U6* and *EF-1α* were used as the internal control in *miR319* and *CnTCP* expression detection, respectively. Values are means ± SD. The different capital letters represent significant differences at *p* < 0.01 according to the Duncan’s test.

A previous study reported the importance of timing and expression levels of *miR319* and its target genes involved in cold tolerance [54,55]. The Arabidopsis *miR319a* and *miR319b* precursor sequences were used to search the *miR319* sequences in the transcriptome of *C. nankingense* (Supplementary Table S1). Due to the two members of *miR319* precursor in *C. nankingense* sharing the same mature sequences with the mature sequence of Arabidopsis *miR319*, 5′ TTGGACTGAAGGGAGCTCCC 3′, this sequence was selected for expression validation and further functional analysis. As shown for other species, mature *miR319* was also predicted to target the expression of *CnTCP2*, *CnTCP4*, and *CnTCP14*. The critical miRNA-target pairing region spans the nucleotides 2–13, which were perfect matches (Figure 6A). Stem-loop qPCR analysis showed that the expression level of mature *miR319* was rapidly upregulated after 6 h of LT treatment, but decreased to control (0 h) level after 24 h (Figure 6B). Some studies have shown the *EF-1α* was sufficient for accurate normalization in cold-treated samples, and the *EF-1α* gene was the frequently-used internal control in the genus chrysanthemum for normalizing transcription data [70,71,72]. When performing microRNA detection, because a better strategy is choosing a constitutively expressed small noncoding RNA as a reference, we often chose the *U6* gene [73,74,75]. The *U6* gene sequence of *C. nankingense* was retrieved using the *AtU6* gene sequence from Arabidopsis (Supplementary Table S1). Interestingly, the expression levels of *CnTCP2*, *CnTCP4,* and *CnTCP14* were significantly downregulated at 6 h at 4 °C, concomitantly with a significant upregulation of *miR319* expression. Similarly, the expression level of *CnTCP4* tended to be upregulated, consistent with the downregulated expression of *miR319* observed at 24 h (Figure 6B). As shown in Figure 6B, there is a significant difference between the expression of *CnTCP2* or *14* with *CnTCP4*. Compared with the expression patterns of *CnTCP2* and *CnTCP14*, which were first downregulated and then stabilized, the trend of first downregulation and then upregulation of *CnTCP4* was more consistent with the expression pattern of *miR319*, indicating that *CnTCP4* may be more dependent on the regulation of *miR319.* Moreover, after one week of cold acclimation, *CnTCP4* was still downregulated compared with the control (Figure 5B), indicating that *CnTCP4* may maintain a continuous function during cold acclimation compared to *CnTCP14* and *CnTCP2.*

### 2.4. CnTCP4 Transcription Factors Are Involved in Cold Stress Response

**Figure 7:**
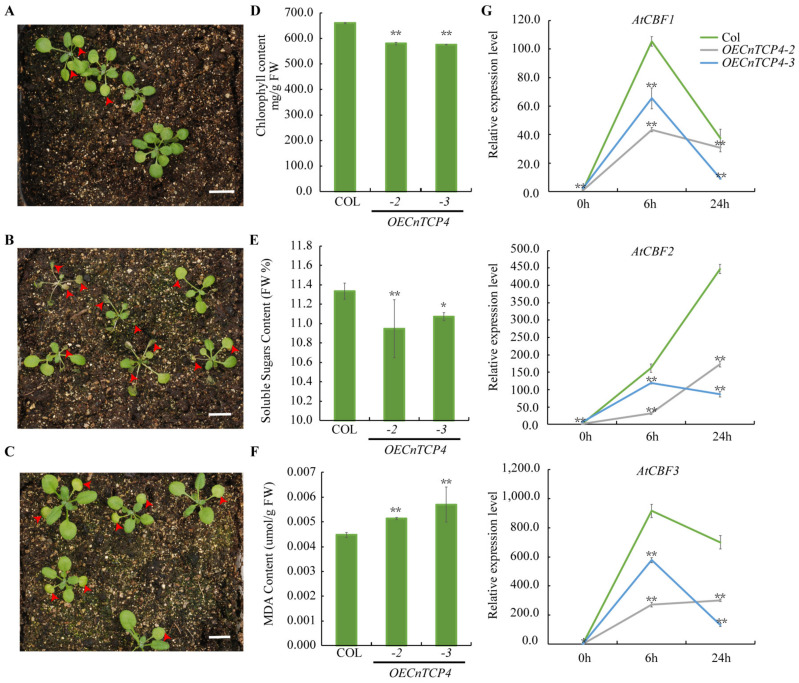
*CnTCP4* negatively regulated cold acclimation. Sensitivity of cold acclimated plants to freezing. (**A**–**C**) show the wildtype Arabidopsis, *CnTCP4-2*, and *CnTCP4-3* overexpressing seedlings after a 2-day recovery from a 20 min freezing treatment at −20 °C, respectively. The leaves with wilting and yellowing phenotypes were labeled by red arrows. (**D**–**F**) show the chlorophyll, soluble sugar, and malondialdehyde (MDA) contents in 4-week-old *CnTCP4* overexpressing Arabidopsis seedlings exposed to 4 °C for two days, respectively. (**G**) 8-day-old seedlings of *OECnTCP4* Arabidopsis were treated at 4 °C for 0, 6, and 24 h. Expression of cold-related genes *AtCBF1*/*2*/*3* were assessed by performing quantitative real-time PCRs. *ACTIN8* was used as the internal control. Values are means ± SD. One and two asterisks represent significant differences at *p* < 0.05 and *p* < 0.01, respectively, according to the Duncan’s test.

Next, we selected *CnTCP4* to test its potential role in cold acclimation using *CnTCP4* overexpressing Arabidopsis lines previously generated by our group [60]. The expression levels of *CnTCP4* in transgenic plants and wildtype are shown in Supplementary Figure S4A, which showed obviously higher expression levels in overexpressing *CnTCP4* Arabidopsis plants. In Figure 7B andC, *OECnTCP4* Arabidopsis showed more wilting and yellowing leaves than the wildtype (Figure 7A) after freezing treatment. Thus, *CnTCP4* overexpressing lines showed higher sensitivity to freezing after cold acclimation. To further determine the mechanism of action of *CnTCP4* on cold tolerance in Arabidopsis, we measured chlorophyll, soluble sugar, and malondialdehyde (MDA) contents in *CnTCP4* overexpressing Arabidopsis after cold acclimation for two days. Compared to wildtype *Arabidopsis thaliana*, *CnTCP4* overexpression decreased chlorophyll (Figure 7D) and soluble sugar contents (Figure 7E), while MDA content increased compared with that in control plants (Figure 7F). Previous studies have reported that expression of *CBF* genes was rapidly induced when Arabidopsis plants were exposed to low temperature (4 °C) [76,77]. Conversely, our findings show that *CnTCP4* negatively regulated the expression of cold-induced genes *AtCBF1*/*2*/*3* (Figure 7G) and the *CBF*-regulated *COR* genes *AtCOR15A* and *AtKIN1* (Supplementary Figure S4B).

## 3. Discussion

The plant-specific TCP family comprises a group of transcription factors that influence cell proliferation and differentiation and consequently regulate growth and developmental processes. Furthermore, TCP family members have been identified in a range of plant species. Although plant-specific *TCP* genes have been thoroughly analyzed in numerous species, systematical analysis of this gene family in *C. nankingense* had not been reported. In this study, phylogenetic and structural analyses of 23 *CnTCP* genes were conducted, and our results revealed the existence of fewer *TCP* genes in *C. nankingense* than in Arabidopsis [5], maize [9], or soybeans [8], suggesting that these few *CnTCPs* might perform multiple functions and be part of gene regulatory networks.

Furthermore, at least in some cases, interaction partners might reportedly alter *TCP* functional specificity [30]. Some interactions with circadian clock-related genes were identified, such as the interactions between TCP7/20/21/22 and TOC1, between TCP18 and FT, and between TCP7/11/22 and CCA1 (Supplementary Figure S3A). These findings indicated that TCPs might play differential roles in flowering regulation. Concomitant binding of some proteins with TCPs to regulate the expression level of target flowering genes has already been reported [30,32,78,79]. Here, we found that *CnTCP4* and *CnTCP13* delayed bolting time in Arabidopsis (Supplementary Figure S5).

In *G. max*, some *GmTCP* genes appeared to respond to multiple stress factors, including heat, drought, and cold. In these cases, *GmTCP* genes apparently contribute to the plant defense response against stress [80]. A similar stress-response function was reported in other legume species [8]. However, to date, few reports have indicated that TCP transcription factors are involved in the response to cold stress [39,55,57]. Previous work suggested that there might be some specialization of *GID1* gene function during seed germination because *GID1a* and *GID1c* transcripts are more strongly induced by cold stratification than *GID1b* transcripts [81]. Additionally, PPI networks revealed GID1A and GID1B interactions with TCPs (Supplementary Figure S3), thus providing new insights into *TCP*-mediated cold response mechanisms. In rice, expression levels of *Osa-MIR319a* and *Osa-MIR319b* are downregulated after 24 h incubation at 4 ℃ [54]. However, *miRNA319* expression in sugarcane reportedly increases after 24 h of cold stress and then returns to the baseline level at 48 h after treatment initiation [55]. Similarly, here, *miRNA319* expression in *C. nankingense* increased after 6 h of cold stress and returned to the baseline level after 24 h of treatment initiation (Figure 6B). This finding suggests that the response of *miRNA319* to cold stress is a dynamic and complex process. Importantly, the corresponding change in the expression of the predicted *miR319*-target gene *CnTCP4* was observed in response to cold tolerance (Figure 6B), which allowed us to infer that *CnTCP4* might be an important gene involved in cold acclimation. Indeed, the overexpression of *CnTCP4* in Arabidopsis negatively regulated cold acclimation by downregulating cold-induced genes such as *AtCBF1*/*2*/*3*, *AtCOR15A*, and *AtKIN1* (Figure 7G and Supplementary Figure S4B) and reducing physiological indicators such as chlorophyll and soluble sugar contents, while increasing MDA content (Figure 7). At the same time, we found that higher *CnTCP4* expression (Supplementary Figure S4A) caused stronger inhibition of the expression of the *CBF* genes (Figure 7G) and higher sensitivity phenotype to cold treatment (Figure 7A–C), suggesting that there may be a dose effect of *CnTCP4* during plant cold tolerance regulation. Cold stress-related marker genes, such as *DREB1*/*CBF* and *DREB2A*, were induced in *Osa-miR319b* overexpressing lines to a significantly greater extent than that in the wildtype; moreover, a great increase in transcript levels of *DREB1A* and *DREB2A* was observed in the *OsPCF6-* and *OsTCP21-RNAi* lines, concomitant with a decrease in overexpressing lines [39]. Therefore, we hypothesized that *TCP* family genes may be involved in the regulation of plant cold tolerance by directly regulating the expression level of downstream cold response-related target genes. All 18 TCPs form a network and interact with each other (Supplementary Figure S3B), consistent with a previous report according to which TCPs form homo- and heterodimers [1,5]. DNA binding seemingly requires dimer formation [40]. Furthermore, homo- and heterodimer interactions show different DNA-binding efficiencies, or mutually modulate their activity, and possibly bind to slightly different *cis*-regulatory elements [82]. However, whether CnTCP4 or its interaction complex binds directly to the cold-related marker genes warrants further research.

Meanwhile, the target *TCP* genes of *miRNA319* might also be regulated by other transcription factors, such as AP2, ERF, bHLH, MYB, WRKY, and *TCP* encoded TFs, which might bind to *cis*-regulatory elements in *CnTCPs* and *AtTCPs* promoters (Supplementary Table S5). Moreover, in Arabidopsis, TCP2 could interact with the cryptochrome 1 (CRY1) protein in yeast and plant cells and be a transcription activator which acts downstream of CRY1 [83]. CRY1 regulates both basal and acquired freezing tolerance in Arabidopsis [84]. The Class I TCP family members with which CnTCP14 was adjacent with CnTCP2 in the evolutionary tree (see Figure 1) also have the same sequence of bHLH domains (see Figure 2) and same protein structures (see Figure 3). Therefore, CnTCP14 may play the same function as CnTCP2. These results suggest that *TCP2* and *TCP14* are not only regulated by *miR319* during cold acclimation, but these members may also be regulated by other transcriptional regulators.

## 4. Materials and Methods

### 4.1. Phylogenetic Analysis of the CnTCP Family

To investigate the phylogenetic relationship of the TCP family, the conserved bHLH-domain amino-acid sequences of CnTCPs identified in *C. nankingense*, *H. annuus*, Arabidopsis, maize, rice, and soybeans were aligned using the multiple sequence alignment program ClustalX. A neighbor-joining (NJ) phylogenetic tree was constructed using the MEGA 7.0 program with the bootstrap value set at 1000 [85]. The *TCP* gene sequences from *C. nankingense* used for analysis were chosen according to previous studies of our group [57,58,59,60,62,86]. The *TCP* gene sequences from Arabidopsis, maize, rice, and soybean used for analysis were chosen according to previous studies [5,8,9]. To identify all TCP proteins in *H. annuus*, all proteins were downloaded from the Sunflower Genome Database (https://www.sunflowergenome.org/, accessed on 18 February 2022) [63]. The Hidden Markov Model (HMM) profile of the TCP domain (PF03634) was downloaded from Protein family (Pfam) (http://pfam.sanger.ac.uk/, accessed on 18 February 2022) and used for identifying TCP proteins from the downloaded database of Sunflower Genome using the HMM search of TBtools [87]. The percent identities among full-length CnTCP proteins from *C. nankingense* or the bHLH motif sequences of TCP proteins from other species were analyzed using the DNAMAN software.

### 4.2. Structural Analysis of the TCP Genes

Information about the gene structure for Arabidopsis *TCP* genes was obtained from the gff3 file GCF_000001735.4_TAIR10.1_genomic downloaded from the website (https://www.ncbi.nlm.nih.gov/genome/browse#!/overview/, accessed on 18 February 2022). All 23 *C. nankingense CnTCP* gene sequences were selected to blast the most homologous genes in the *C. nankingense* genome scaffolds and protein files (http://www.amwayabrc.com/, accessed on 18 February 2022) for searching the accession numbers. For *C. nankingense TCP* genes, information about the gene structures was procured from the gff3 file Chrysanthemum_genome_gene_v2.0 using the searched accession numbers. The TBtools software was used to draw the gene structure diagram [87].

Protein sequences were analyzed in the MEME program (http://meme.sdsc.edu/meme/cgi-bin/meme.cgi, accessed on 18 February 2022) to confirm the conserved motifs. The MEME program was used under the following parameters: zero or one occurrence per sequence (zoops), maximum number of motifs—4, and optimum motif width set to >6 and <50. The psRNATarget website was used for *miR319* target gene prediction (https://www.zhaolab.org/psRNATarget/, accessed on 18 February 2022). The mature sequences of *miR319* and all *CnTCPs* family sequences were used for target site analyses.

### 4.3. Protein–Protein Interaction Network Analysis

Protein–protein interaction networks of CnTCPs were analyzed using the search tool for Retrieval of Interacting Genes (STRING; http://stringdb. org/, accessed on 18 February 2022) online. The screened networks were visualized by Cytoscape 3.9.0. The MCODE software was used to establish PPI network modules using parameters of degree cutoff 2, k-core 2, max. depth 100, and node score cutoff 0.2.

### 4.4. Putative Cis-Elements in the Promoter Regions

The 3000-bp upstream sequences from the translation start codon of all *CnTCP* family genes were obtained from the *C. nankingense* Genome scaffolds files (http://www.amwayabrc.com/, accessed on 18 February 2022). The cis-elements of promoters were identified using the PlantRegMap: Binding Site Prediction (http://plantregmap.gao-lab.org/, accessed on 18 February 2022) [88] and PlantCARE: Search for CARE [89].

### 4.5. Plant Growth Conditions and Cold Treatment

Diploid *C. nankingense* was obtained from the Chrysanthemum Germplasm Resource Preserving Center at Nanjing Agricultural University, China. Plants were grown under a 22/15 °C day/night temperature conditions, at a relative humidity of 70–75%, and a 16/8 h (day/night) photoperiod. For cold stress, plants were subjected to 4 °C during the day/night and a 16/8 h photoperiod environment. *CnTCP4* overexpressing Arabidopsis was previously generated by our group [22,60]. For the freezing test, four-week-old plants were cold-acclimated (at 4 °C) for two days and then subjected to freezing at −20 °C for 20 min; finally, they were observed after a 2 d recovery at 23°C. Chlorophyll, soluble sugar, and malondialdehyde (MDA) contents in *OECnTCP4* Arabidopsis were measured after cold stress according to the methods previously described [90,91,92]. Samples were harvested without any additional treatment. For each treatment, aboveground plant biomass was harvested. For cold treatment shown in Figure 6 and Figure S4B, the plant part of two-month old plants of *C. nankingense* from the apical bud to the third fully expanded leaf were harvested.

### 4.6. Verification of CnTCP Expression

Total RNA was extracted from each sample. After digestion with RNase-free DNase I (TaKaRa; Tokyo, Japan) and reverse transcription with Reverse Transcription M-MLV (TaKaRa), about 100 ng cDNA product was used for each reaction (10 μL 2× SYBR^®^ Premix Ex TaqTM II and 0.5 μL of 10 μM primer pairs mixed in a 20 μL reaction system). DNA was extracted from the *OECnTCP4* and wildtype Arabidopsis seedlings using the CTAB method. The chrysanthemum *EF-1α* gene (Genbank accession number KF305681) and Arabidopsis *ACTIN8* gene were used as reference genes, respectively. Primers used in this study are listed in Supplementary Table S1.

## 5. Conclusions

Here, we identified 23 *CnTCP* genes in *C. nankingense*, systematically analyzed their phylogenetic relationships and synteny with TCPs from other species, and evaluated their expression profiles at low temperature. A total of 23 CnTCP proteins fall into two classes and three clades, with a typical bHLH domain. Predicted protein structure and binding sites analysis suggested a unique function of CnTCPs in *C. nankingense*. Expression of most *CnTCPs* were downregulated under cold conditions, suggesting their importance in plant responses to cold stress. Notably, expression of *miR319* and of its predicted target genes, *CnTCP2*/*4*/*14*, led to fast responses to cold. Overexpression of *CnTCP4* in Arabidopsis led to hypersensitivity to cold, suggesting that *CnTCP4* might play a negative role in *C. nankingense* responses to cold stress. Our results provide a foundation for future functional genomic studies on this gene family in chrysanthemum.

## Figures and Tables

**Table 1:** Percentage homology among full-length CnTCPs proteins.

CnTCPS	CnTCP4-1	CnTCP4-3	CnTCP4-2	CnTCP14	CnTCP2-3	CnTCP2-2	CnTCP2-1	CnTCP13	CnTCP5	CnTCP17	CnTCP3	CnTCP15
CnTCP10	10.70%	11.40%	11.00%	11.60%	11.80%	12.10%	12.10%	10.30%	12.90%	16.30%	12.30%	11.20%
CnTCP12	11.20%	11.90%	11.20%	12.40%	11.30%	11.40%	11.30%	10.60%	14.20%	15.50%	12.00%	11.20%
CnTCP16	14.10%	14.20%	14.10%	13.10%	15.50%	15.50%	15.50%	12.90%	13.80%	16.10%	15.50%	15.20%
CnTCP9	15.80%	15.70%	15.70%	11.80%	12.60%	12.70%	12.70%	13.20%	18.80%	17.60%	15.10%	14.40%
CnTCP7	15.30%	14.80%	14.80%	14.70%	14.80%	15.00%	14.80%	12.00%	14.20%	13.80%	15.50%	16.80%
CnTCP8	14.00%	13.40%	13.70%	9.40%	11.00%	11.10%	11.00%	12.20%	12.90%	12.80%	17.40%	14.10%
CnTCP18-1	11.80%	11.90%	11.80%	13.40%	13.60%	13.60%	13.80%	14.20%	15.50%	18.10%	16.40%	16.10%
CnTCP18-2	12.30%	12.40%	12.30%	14.00%	13.70%	13.70%	14.00%	14.70%	16.00%	17.80%	16.50%	16.20%
CnTCP6	16.40%	16.10%	16.40%	15.70%	14.90%	14.70%	14.60%	15.80%	17.60%	17.40%	14.80%	15.80%
CnTCP1	19.80%	20.60%	20.60%	19.10%	17.60%	17.60%	17.50%	17.50%	19.70%	22.50%	31.10%	29.20%
CnTCP11	20.70%	21.50%	20.80%	15.70%	17.40%	17.40%	17.30%	17.30%	14.90%	19.10%	27.00%	25.90%
CnTCP15	16.00%	16.50%	16.00%	17.60%	18.80%	18.80%	18.70%	16.00%	16.20%	19.90%	46.20%	100%
CnTCP3	16.50%	15.70%	16.10%	15.40%	17.00%	17.00%	16.90%	16.70%	17.80%	17.90%	100%	
CnTCP17	20.10%	20.30%	20.40%	25.30%	26.20%	26.10%	26.00%	37.20%	52.10%	100%		
CnTCP5	19.50%	20.10%	19.50%	23.90%	23.70%	23.70%	23.60%	34.70%	100%			
CnTCP13	19.90%	21.00%	21.00%	26.60%	24.60%	24.60%	24.80%	100%				
CnTCP2-1	20.20%	19.40%	20.00%	57.40%	99.50%	99.80%	100%					
CnTCP2-2	20.30%	19.50%	20.10%	57.10%	99.80%	100%						
CnTCP2-3	20.40%	19.60%	20.10%	57.10%	100%							
CnTCP14	22.50%	21.90%	22.40%	100%								
CnTCP4-2	93.80%	96.00%	100%									
CnTCP4-3	90.10%	100%										
CnTCP4-1	100%											
**CnTCPS**	**CnTCP11**	**CnTCP1**	**CnTCP6**	**CnTCP18-2**	**CnTCP18-1**	**CnTCP8**	**CnTCP7**	**CnTCP9**	**CnTCP16**	**CnTCP12**	**CnTCP10**	
CnTCP10	12.40%	11.90%	26.80%	28.00%	28.30%	34.20%	28.70%	36.00%	50.00%	68.10%	100%	
CnTCP12	11.60%	13.30%	26.40%	28.40%	28.60%	34.00%	29.10%	34.00%	50.60%	100%		
CnTCP16	11.50%	13.80%	27.40%	31.30%	31.70%	38.10%	35.70%	39.30%	100%			
CnTCP9	14.80%	18.20%	28.70%	31.50%	31.50%	28.90%	33.10%	100%				
CnTCP7	16.20%	16.20%	28.80%	30.20%	29.20%	38.50%	100%					
CnTCP8	13.00%	14.50%	28.80%	29.70%	29.20%	100%						
CnTCP18-1	14.10%	16.20%	41.70%	97.30%	100%							
CnTCP18-2	14.20%	16.20%	41.30%	100%								
CnTCP6	16.30%	13.90%	100%									
CnTCP1	52.30%	100%										
CnTCP11	100%											

Relatively high identities >50% were displayed in red font.

**Table 2:** Statistical analysis of TFs identified from *CnTCP* promoter sequences.

Family	AtTCP	CnTCP	Family	AtTCP	CnTCP
AP2	24	18	TCP	22	17
B3	24	18	ARF	24	16
bHLH	24	18	BBR-BPC	24	16
bZIP	24	18	WRKY	24	16
C2H2	24	18	ZF-HD	22	16
Dof	24	18	LBD	21	16
ERF	24	18	SBP	21	16
G2-like	24	18	C3H	20	16
GATA	24	18	WOX	22	15
HD-ZIP	24	18	CPP	21	15
MIKC_MADS	24	18	EIL	18	15
MYB	24	18	NF-YB	16	15
MYB_related	24	18	E2F/DP	23	14
NAC	24	18	Nin-like	22	14
Trihelix	23	18	RAV	22	14
GRAS	23	17	SRS	21	13
			ARR-B	20	7

Data indicate the number of TCP genes with predicted TFs.

## Data Availability

Not applicable.

## Supplementary Materials

The following supporting information can be downloaded at: https://www.mdpi.com/article/10.3390/plants11070936/s1, Figure S1. Evolutionary tree of TCP proteins; Figure S2. Gene structure of TCPs; Figure S3. Co-expression network analysis of TCP proteins and correlated proteins; Figure S4. Expression of cold-related genes after cold stress and identification of OECnTCP4; Figure S5. Overexpression of CnTCP4 and CnTCP13 delayed bolting in Arabidopsis thaliana; Table S1. Primers and sequences used in this research; Table S2. Distance and homology matrices of 171 sequences were generated with the DNAMAN program using the full-length amino acid sequences from Glycine max, Oryza sativa, Zea mays, Chrysanthemum nankingense, and Arabidopsis thaliana; Table S3. The distance and homology matrix of 171 sequences were generated with the DNAMAN program using the bHLH amino acid sequences from Glycine max, Oryza sativa, Zea mays, Chrysanthemum nankingense, and Arabidopsis thaliana; Table S4. Distance and homology matrices of 23 sequences were generated with the DNAMAN program using the full-length and bHLH amino acid sequences from Chrysanthemum nankingense; Table S5. Binding site prediction results of TCP promoters from Chrysanthemum nankingense and Arabidopsis thaliana using the PlantRegMap website; Table S6. Cis-acting regulatory element prediction results of TCP promoters from Chrysanthemum nankingense and Arabidopsis thaliana using the PlantCARE website.
