# Supplementary material for: Characterization of the TCP Gene Family in Chrysanthemum nankingense and the Role of CnTCP4 in Cold Tolerance
Source: Plants (Basel). 2022 Mar 30;11(7):936. doi: 10.3390/plants11070936 (PMC9002959; doi:10.3390/plants11070936)
Supplement: Supplementary file 1 [file plants-11-00936-s001.zip › Supplementary Table S6/Supplement S6 PlantCARE_5042_CnTCP PROMOTER.pdf]

Motifs Found

- +
- + A-box
- + AAGAA-motif
- + ABRE
- + ABRE3a
- + ABRE4
- + ARE
- + ATCT-motif
- + AT~TATA-box
- + Box 4
- + CAAT-box
- + CCGTCC motif
- + CCGTCC-box
- + CGTCA-motif
- + CTAG-motif
- + DRE1
- + G-box
- + GATA-motif
- + GCN4_motif
- + GT1-motif
- + I-box
- + LAMP-element
- + MBS
- + MRE
- + MYB
- + MYB-like sequence
- + MYC
- + Myb
- + Myb-binding site
- + O2-site
- + P-box
- + TATA
- + TATA-box
- + TCA
- + TCA-element
- + TCCC-motif
- + TGACG-motif
- + Unnamed__1
- + Unnamed__16
- + Unnamed__4
- + Unnamed__6
- + W box

+		WRE3
+		WUN-motif
+		Y-box
+		as-1

Motifs Found

+
+ 4cl-CMA2b
+ AAGAA-motif
+ ABRE
+ ABRE3a
+ ABRE4
+ ARE
+ AT-rich sequence
+ ATC-motif
+ AT~TATA-box
+ Box 4
+ CAAT-box
+ CARE
+ CAT-box
+ CGTCA-motif
+ G-box
+ GC-motif
+ GCN4_motif
+ GT1-motif
+ LAMP-element
+ LTR
+ MBS
+ MBSI
+ MRE
+ MYB
+ MYB-like sequence
+ MYC
+ Myb
+ Myb-binding site
+ Myc
+ STRE
+ TATA-box
+ TC-rich repeats
+ TCA-element
+ TCT-motif
+ TGACG-motif
+ Unnamed__1
+ Unnamed__2
+ Unnamed__4
+ W box
+ WUN-motif
+ as-1

+ chs-Unit 1 m1

Motifs Found

- +
- + AAGAA-motif
- + ABRE
- + ARE
- + ATC-motif
- + AT~TATA-box
- + Box 4
- + Box III
- + CAAT-box
- + CGTCA-motif
- + DRE core
- + ERE
- + G-Box
- + G-box
- + GARE-motif
- + GT1-motif
- + I-box
- + MRE
- + MYB
- + MYB-like sequence
- + MYC
- + Myb
- + Myb-binding site
- + O2-site
- + STRE
- + TATA-box
- + TATC-box
- + TC-rich repeats
- + TCA-element
- + TCCC-motif
- + TGACG-motif
- + Unnamed__1
- + Unnamed__4
- + Unnamed__6
- + W box
- + WRE3
- + as-1
- + box S
- + chs-CMA1a

Motifs Found

+
+ A-box
+ AAGAA-motif
+ ABRE
+ AC-I
+ ARE
+ ATCT-motif
+ AT~TATA-box
+ Box 4
+ CAAT-box
+ CAT-box
+ CCAAT-box
+ CCGTCC motif
+ CCGTCC-box
+ CGTCA-motif
+ ERE
+ G-Box
+ GA-motif
+ GATA-motif
+ GT1-motif
+ LTR
+ MRE
+ MYB
+ MYB recognition site
+ MYB-like sequence
+ Myc
+ O2-site
+ P-box
+ STRE
+ TATA
+ TATA-box
+ TC-rich repeats
+ TCT-motif
+ TGA-element
+ TGACG-motif
+ Unnamed__2
+ Unnamed__4
+ W box
+ WRE3
+ WUN-motif
+ as-1
+ box S

\+ chs-Unit 1 m1

Motifs Found

- +
- + AAGAA-motif
- + AE-box
- + AP-1
- + ARE
- + AT~TATA-box
- + Box 4
- + Box II -like sequence
- + CAAT-box
- + CAT-box
- + CGTCA-motif
- + CTAG-motif
- + DRE core
- + GT1-motif
- + MBS
- + MRE
- + MYB
- + MYB-like sequence
- + MYC
- + Myb
- + P-box
- + STRE
- + TATA-box
- + TATC-box
- + TCCC-motif
- + TCT-motif
- + TGA-element
- + TGACG-motif
- + Unnamed__1
- + Unnamed__4
- + W box
- + WRE3
- + WUN-motif
- + as-1
- + box S
- + chs-CMA1a
- + circadian

Motifs Found

- +
- + 3-AF3 binding site
- + 4cl-CMA1b
- + AAGAA-motif
- + ABRE
- + ABRE3a
- + ABRE4
- + ACE
- + ARE
- + AT-rich sequence
- + AT~TATA-box
- + Box 4
- + CAAT-box
- + CAT-box
- + CGTCA-motif
- + DRE core
- + ERE
- + G-Box
- + G-box
- + GATA-motif
- + GCN4_motif
- + GT1-motif
- + LTR
- + MRE
- + MSA-like
- + MYB
- + MYB-like sequence
- + MYC
- + STRE
- + TATA
- + TATA-box
- + TC-rich repeats
- + TCA
- + TCA-element
- + TGACG-motif
- + Unnamed__1
- + Unnamed__4
- + Unnamed__6
- + as-1
- + dOCT

Motifs Found

+
+ AAGAA-motif
+ ABRE
+ ABRE3a
+ ABRE4
+ AE-box
+ ARE
+ ATC-motif
+ AT~ABRE
+ AT~TATA-box
+ CAAT-box
+ CAT-box
+ CGTCA-motif
+ DRE core
+ DRE1
+ G-box
+ GA-motif
+ GT1-motif
+ Gap-box
+ I-box
+ LTR
+ MBS
+ MRE
+ MYB
+ MYB-like sequence
+ MYC
+ Myb
+ Myb-binding site
+ O2-site
+ P-box
+ STRE
+ TATA-box
+ TC-rich repeats
+ TCA
+ TCT-motif
+ TGA-element
+ TGACG-motif
+ Unnamed__1
+ Unnamed__4
+ W box
+ WRE3
+ WUN-motif

+		as-1
+		circadian

Motifs Found

- +
- + A-box
- + AAGAA-motif
- + ABRE
- + ABRE3a
- + ABRE4
- + AC-II
- + ARE
- + AT-rich element
- + AT~TATA-box
- + Box 4
- + CAAT-box
- + CAT-box
- + CCGTCC motif
- + CCGTCC-box
- + G-Box
- + G-box
- + GA-motif
- + GC-motif
- + GT1-motif
- + LAMP-element
- + LTR
- + MRE
- + MYB
- + MYB-like sequence
- + MYC
- + Myb
- + P-box
- + STRE
- + TATA
- + TATA-box
- + TATC-box
- + TCA-element
- + TCT-motif
- + TGA-element
- + Unnamed__1
- + Unnamed__2
- + Unnamed__4
- + W box
- + WRE3
- + WUN-motif
- + box S

\+ chs-CMA1a

Motifs Found

+
+ AAGAA-motif
+ ABRE
+ ARE
+ AT~TATA-box
+ Box 4
+ CAAT-box
+ CAT-box
+ CGTCA-motif
+ ERE
+ G-Box
+ G-box
+ GA-motif
+ GATA-motif
+ GT1-motif
+ LTR
+ MRE
+ MYB
+ MYB-like sequence
+ MYC
+ Myb
+ P-box
+ TATA
+ TATA-box
+ TC-rich repeats
+ TCA
+ TGACG-motif
+ Unnamed__1
+ Unnamed__2
+ Unnamed__4
+ Unnamed__6
+ W box
+ WUN-motif
+ as-1
+ circadian

Motifs Found

+		
+		AAGAA-motif
+		ABRE
+		AP-1
+		ARE
+		AT-rich element
+		Box 4
+		CAAT-box
+		CAT-box
+		CGTCA-motif
+		DRE1
+		ERE
+		G-Box
+		G-box
+		GATA-motif
+		GT1-motif
+		LTR
+		MBS
+		MBSI
+		MYB
+		MYB-like sequence
+		MYC
+		Myb
+		Myb-binding site
+		O2-site
+		P-box
+		STRE
+		TATA-box
+		TCA
+		TGACG-motif
+		Unnamed__1
+		Unnamed__4
+		Unnamed__6
+		WUN-motif
+		as-1

Motifs Found

- +
- + A-box
- + ABRE
- + ACE
- + ARE
- + AT-rich element
- + ATCT-motif
- + AT~TATA-box
- + Box 4
- + CAAT-box
- + CCAAT-box
- + CCGTCC motif
- + CCGTCC-box
- + DRE1
- + ERE
- + G-Box
- + G-box
- + GATA-motif
- + GT1-motif
- + I-box
- + LTR
- + MRE
- + MYB
- + MYB recognition site
- + MYC
- + Myb
- + Myb-binding site
- + STRE
- + TATA-box
- + TATC-box
- + TCT-motif
- + TGA-element
- + Unnamed__4
- + Unnamed__6
- + WRE3
- + WUN-motif
- + chs-CMA1a

Motifs Found

- +
- + AAGAA-motif
- + ABRE
- + ABRE3a
- + ABRE4
- + ACE
- + AE-box
- + ARE
- + AT~TATA-box
- + AuxRR-core
- + Box 4
- + Box II
- + CAAT-box
- + CGTCA-motif
- + ERE
- + G-box
- + GARE-motif
- + GATA-motif
- + GT1-motif
- + LTR
- + MBS
- + MRE
- + MYB
- + MYB-like sequence
- + MYC
- + Myb
- + Myb-binding site
- + O2-site
- + RY-element
- + STRE
- + TATA-box
- + TC-rich repeats
- + TCA
- + TCT-motif
- + TGA-element
- + TGACG-motif
- + Unnamed__1
- + Unnamed__10
- + Unnamed__12
- + Unnamed__14
- + Unnamed__4
- + Unnamed__8

+		W box
+		WRE3
+		as-1
+		box S
+		chs-CMA2a

Motifs Found

+
+ 3-AF1 binding site
+ A-box
+ AAGAA-motif
+ ABRE
+ ABRE3a
+ ABRE4
+ AE-box
+ ARE
+ AT-rich sequence
+ ATC-motif
+ AT~TATA-box
+ AuxRR-core
+ Box 4
+ Box III
+ CAAT-box
+ CCGTCC motif
+ CCGTCC-box
+ CGTCA-motif
+ ERE
+ F-box
+ G-Box
+ G-box
+ GA-motif
+ GATA-motif
+ GCN4_motif
+ GT1-motif
+ HD-Zip 3
+ I-box
+ LTR
+ MBS
+ MRE
+ MYB
+ MYB-like sequence
+ MYC
+ Myb
+ Myb-binding site
+ O2-site
+ P-box
+ STRE
+ TATA
+ TATA-box

+		TCA-element
+		TCT-motif
+		TGA-box
+		TGA-element
+		TGACG-motif
+		Unnamed__1
+		Unnamed__2
+		Unnamed__4
+		W box
+		WUN-motif
+		as-1
+		chs-CMA1a
+		circadian

Motifs Found

+

+ AAGAA-motif

+ ABRE

+ ABRE3a

+ ABRE4

+ ARE

+ AT~TATA-box

+ Box 4

+ CAAT-box

+ CGTCA-motif

+ DRE core

+ G-box

+ GA-motif

+ GATA-motif

+ GCN4_motif

+ GT1-motif

+ LAMP-element

+ MBS

+ MBSI

+ MRE

+ MYB

+ MYB-like sequence

+ MYC

+ Myb

+ P-box

+ TATA

+ TATA-box

+ TCA

+ TCA-element

+ TGA-element

+ TGACG-motif

+ Unnamed__1

+ Unnamed__4

+ W box

+ as-1

+ box S

+ chs-CMA1a

Motifs Found

- +
- + AAGAA-motif
- + ABRE
- + ABRE3a
- + ABRE4
- + AT-rich sequence
- + AT~TATA-box
- + AuxRR-core
- + Box 4
- + CAAT-box
- + CAT-box
- + CCAAT-box
- + CGTCA-motif
- + G-Box
- + G-box
- + GATA-motif
- + GT1-motif
- + MBS
- + MYB
- + MYB recognition site
- + MYB-like sequence
- + MYC
- + Myb
- + Myb-binding site
- + P-box
- + STRE
- + TATA-box
- + TCA
- + TCA-element
- + TCT-motif
- + TGA-element
- + TGACG-motif
- + Unnamed__1
- + Unnamed__4
- + Unnamed__6
- + W box
- + WUN-motif
- + as-1
- + chs-CMA1a

Motifs Found

- +
- + ABRE
- + ABRE3a
- + ABRE4
- + AC-I
- + AC-II
- + ARE
- + AT-rich sequence
- + ATCT-motif
- + AT~TATA-box
- + Box 4
- + Box II -like sequence
- + CAAT-box
- + CARE
- + CGTCA-motif
- + CTAG-motif
- + ERE
- + G-Box
- + G-box
- + GARE-motif
- + GT1-motif
- + Gap-box
- + MBS
- + MRE
- + MYB
- + MYC
- + Myb
- + Myb-binding site
- + O2-site
- + P-box
- + SARE
- + STRE
- + TATA
- + TATA-box
- + TC-rich repeats
- + TCA
- + TCA-element
- + TCCC-motif
- + TCT-motif
- + TGACG-motif
- + Unnamed__1
- + Unnamed__4

+ WRE3
+ WUN-motif
+ as-1
+ chs-Unit 1 m1

Motifs Found

+
+ AAGAA-motif
+ ABRE
+ ABRE3a
+ ABRE4
+ ARE
+ AT~TATA-box
+ Box 4
+ CAAT-box
+ CAT-box
+ CGTCA-motif
+ DRE core
+ G-Box
+ G-box
+ GA-motif
+ GARE-motif
+ GATA-motif
+ I-box
+ LAMP-element
+ MBS
+ MYB
+ MYB-like sequence
+ MYC
+ Myb
+ Myb-binding site
+ Myc
+ P-box
+ RY-element
+ STRE
+ TATA-box
+ TCT-motif
+ TGACG-motif
+ Unnamed__1
+ Unnamed__4
+ as-1
+ circadian

Motifs Found

+	
+	ABRE
+	AE-box
+	ARE
+	AT~TATA-box
+	AuxRR-core
+	Box 4
+	CAAT-box
+	CCAAT-box
+	CGTCA-motif
+	ERE
+	F-box
+	G-box
+	GA-motif
+	GATA-motif
+	GT1-motif
+	LTR
+	MBS
+	MRE
+	MYB
+	MYB recognition site
+	MYB-like sequence
+	MYC
+	Myb
+	STRE
+	TATA-box
+	TC-rich repeats
+	TCA
+	TCT-motif
+	TGA-box
+	TGACG-motif
+	Unnamed__1
+	Unnamed__4
+	W box
+	WRE3
+	WUN-motif
+	as-1
+	re2f-1
