# Supplementary material for: Characterization of the TCP Gene Family in Chrysanthemum nankingense and the Role of CnTCP4 in Cold Tolerance
Source: Plants (Basel). 2022 Mar 30;11(7):936. doi: 10.3390/plants11070936 (PMC9002959; doi:10.3390/plants11070936)
Supplement: Supplementary file 1 [file plants-11-00936-s001.zip › Supplementary Table S6/Supplement S6 PlantCARE_8936_AtTCP PROMOTER.pdf]

Motifs Found

- +
- + AAGAA-motif
- + AE-box
- + ARE
- + AT-rich sequence
- + AT~TATA-box
- + Box 4
- + CAAT-box
- + CAT-box
- + CGTCA-motif
- + ERE
- + GCN4_motif
- + GT1-motif
- + MBS
- + MYB
- + MYB-like sequence
- + MYC
- + Myb
- + Myc
- + O2-site
- + TATA
- + TATA-box
- + TCA
- + TCA-element
- + TCT-motif
- + TGA-element
- + TGACG-motif
- + Unnamed__4
- + Unnamed__6
- + W box
- + WRE3
- + WUN-motif
- + as-1
- + circadian

Motifs Found

- +
- + AAGAA-motif
- + AE-box
- + ARE
- + AT~TATA-box
- + Box 4
- + CAAT-box
- + CGTCA-motif
- + ERE
- + GA-motif
- + GT1-motif
- + Gap-box
- + LAMP-element
- + LTR
- + MBS
- + MYB
- + MYB-like sequence
- + MYC
- + Myb
- + Myb-binding site
- + STRE
- + TATA
- + TATA-box
- + TC-rich repeats
- + TCA-element
- + TCT-motif
- + TGA-element
- + TGACG-motif
- + Unnamed__1
- + Unnamed__4
- + WRE3
- + WUN-motif
- + as-1

Motifs Found

- +
- + AAGAA-motif
- + ABRE
- + ABRE3a
- + ABRE4
- + ARE
- + AT~TATA-box
- + Box 4
- + Box II
- + CAAT-box
- + CGTCA-motif
- + DRE core
- + ERE
- + G-box
- + GATA-motif
- + GCN4_motif
- + HD-Zip 1
- + I-box
- + LAMP-element
- + LTR
- + MYB
- + MYB-like sequence
- + MYC
- + Myc
- + P-box
- + STRE
- + TATA
- + TATA-box
- + TC-rich repeats
- + TCA
- + TCT-motif
- + TGACG-motif
- + Unnamed__1
- + Unnamed__4
- + W box
- + WRE3
- + as-1

Motifs Found

+		
+		AAGAA-motif
+		ABRE
+		ABRE3a
+		ABRE4
+		ACE
+		ARE
+		AT~TATA-box
+		Box 4
+		CAAT-box
+		CCAAT-box
+		CGTCA-motif
+		DRE1
+		ERE
+		G-box
+		GT1-motif
+		MBS
+		MBSI
+		MRE
+		MYB
+		MYB recognition site
+		MYB-like sequence
+		Myb
+		Myc
+		P-box
+		TATA-box
+		TCA
+		TCCC-motif
+		TCT-motif
+		TGA-element
+		TGACG-motif
+		Unnamed__1
+		Unnamed__4
+		Unnamed__6
+		W box
+		WRE3
+		WUN-motif
+		as-1
+		telo-box

Motifs Found

- +
- + AAGAA-motif
- + ABRE
- + ACE
- + AP-1
- + ARE
- + AT~TATA-box
- + AuxRR-core
- + Box 4
- + CAAT-box
- + CAT-box
- + CGTCA-motif
- + ERE
- + G-box
- + GT1-motif
- + I-box
- + LTR
- + MBSI
- + MYB
- + MYB-like sequence
- + MYC
- + Myb
- + Myb-binding site
- + Myc
- + TATA
- + TATA-box
- + TC-rich repeats
- + TCA
- + TCT-motif
- + TGA-element
- + TGACG-motif
- + Unnamed__4
- + W box
- + as-1

Motifs Found

+	
+	AAGAA-motif
+	ABRE
+	ACE
+	AE-box
+	ARE
+	AT-rich element
+	AT~TATA-box
+	CAAT-box
+	CGTCA-motif
+	G-Box
+	G-box
+	GARE-motif
+	GATA-motif
+	I-box
+	LTR
+	MBS
+	MYB
+	MYB-like sequence
+	MYC
+	Myb
+	Myb-binding site
+	Myc
+	O2-site
+	STRE
+	TATA-box
+	TATC-box
+	TC-rich repeats
+	TCA
+	TCA-element
+	TCT-motif
+	TGACG-motif
+	Unnamed__1
+	Unnamed__4
+	W box
+	WRE3
+	as-1
+	chs-CMA2a
+	circadian

Motifs Found

- +
- + ABRE
- + ABRE3a
- + ABRE4
- + AC-I
- + AE-box
- + ARE
- + AT~TATA-box
- + Box 4
- + Box II
- + CAAT-box
- + CCAAT-box
- + CGTCA-motif
- + DRE core
- + ERE
- + F-box
- + G-Box
- + G-box
- + LTR
- + MBS
- + MRE
- + MYB
- + MYB recognition site
- + MYB-like sequence
- + MYC
- + Myb
- + O2-site
- + P-box
- + STRE
- + TATA
- + TATA-box
- + TC-rich repeats
- + TCA-element
- + TGA-element
- + TGACG-motif
- + Unnamed__1
- + Unnamed__2
- + Unnamed__4
- + Unnamed__6
- + W box
- + WRE3
- + WUN-motif

+ as-1

Motifs Found

- +
- + AAGAA-motif
- + ABRE
- + AE-box
- + ARE
- + AT-rich element
- + AT~TATA-box
- + CAAT-box
- + CGTCA-motif
- + ERE
- + G-box
- + GARE-motif
- + GC-motif
- + I-box
- + LTR
- + MBS
- + MRE
- + MYB
- + MYB-like sequence
- + MYC
- + Myb
- + Myb-binding site
- + STRE
- + TATA-box
- + TCA
- + TCT-motif
- + TGA-element
- + TGACG-motif
- + Unnamed__1
- + Unnamed__2
- + Unnamed__4
- + W box
- + WRE3
- + WUN-motif
- + as-1
- + circadian

Motifs Found

- +
- + A-box
- + AAGAA-motif
- + ABRE
- + ARE
- + ATC-motif
- + ATCT-motif
- + AT~TATA-box
- + AuxRR-core
- + Box 4
- + CAAT-box
- + CAT-box
- + CCAAT-box
- + CCGTCC motif
- + CCGTCC-box
- + CGTCA-motif
- + F-box
- + G-Box
- + G-box
- + GATA-motif
- + GC-motif
- + GT1-motif
- + I-box
- + LTR
- + MRE
- + MYB
- + MYB recognition site
- + MYB-like sequence
- + MYC
- + Myb
- + Myb-binding site
- + O2-site
- + STRE
- + TATA-box
- + TCT-motif
- + TGACG-motif
- + Unnamed__1
- + Unnamed__2
- + Unnamed__4
- + W box
- + WRE3
- + as-1

+ box S
+ re2f-1

Motifs Found

- +
- + AAGAA-motif
- + ABRE
- + ABRE2
- + ARE
- + AT~TATA-box
- + Box 4
- + CAAT-box
- + CAT-box
- + CCAAT-box
- + CGTCA-motif
- + DRE core
- + DRE1
- + G-Box
- + G-box
- + GT1-motif
- + I-box
- + LTR
- + MYB
- + MYB recognition site
- + MYB-like sequence
- + MYC
- + Myb
- + Myb-binding site
- + P-box
- + STRE
- + TATA-box
- + TCA-element
- + TCCC-motif
- + TCT-motif
- + TGACG-motif
- + Unnamed__1
- + Unnamed__10
- + Unnamed__12
- + Unnamed__14
- + Unnamed__4
- + Unnamed__8
- + WRE3
- + as-1

Motifs Found

- +
- + 3-AF1 binding site
- + ABRE
- + ARE
- + Box 4
- + CAAT-box
- + CGTCA-motif
- + G-Box
- + G-box
- + GARE-motif
- + GT1-motif
- + I-box
- + LAMP-element
- + MBS
- + MYB
- + MYC
- + Myb
- + Myb-binding site
- + Myc
- + O2-site
- + P-box
- + STRE
- + TATA-box
- + TATC-box
- + TC-rich repeats
- + TCA
- + TCCC-motif
- + TCT-motif
- + TGACG-motif
- + Unnamed__1
- + Unnamed__2
- + Unnamed__4
- + Unnamed__6
- + WRE3
- + as-1

Motifs Found

- +
- + 3-AF1 binding site
- + AAGAA-motif
- + ABRE
- + ARE
- + AT-rich element
- + AT~TATA-box
- + Box 4
- + Box III
- + CAAT-box
- + CAT-box
- + CGTCA-motif
- + G-Box
- + G-box
- + GA-motif
- + GATA-motif
- + GT1-motif
- + I-box
- + MYB
- + MYB-like sequence
- + MYC
- + Myb
- + Myc
- + NON
- + O2-site
- + P-box
- + STRE
- + TATA
- + TATA-box
- + TCA
- + TCA-element
- + TGA-element
- + TGACG-motif
- + Unnamed__1
- + Unnamed__4
- + Unnamed__6
- + WUN-motif
- + as-1
- + chs-CMA1a

Motifs Found

- +
- + 3-AF1 binding site
- + A-box
- + AAGAA-motif
- + ABRE
- + ABRE2
- + ABRE3a
- + ABRE4
- + AE-box
- + ARE
- + AT-rich element
- + AT~TATA-box
- + AuxRR-core
- + Box 4
- + Box II
- + Box III
- + CAAT-box
- + CCGTCC motif
- + CCGTCC-box
- + CGTCA-motif
- + CTAG-motif
- + ERE
- + G-Box
- + G-box
- + GCN4_motif
- + GT1-motif
- + LAMP-element
- + LTR
- + MYB
- + MYB-like sequence
- + MYC
- + Myb
- + Myc
- + O2-site
- + P-box
- + STRE
- + TATA
- + TATA-box
- + TCA
- + TCT-motif
- + TGA-element
- + TGACG-motif

+		Unnamed__1
+		Unnamed__4
+		Unnamed__6
+		W box
+		as-1

Motifs Found

- +
- + A-box
- + ABRE
- + ABRE3a
- + ABRE4
- + AE-box
- + ARE
- + AT~TATA-box
- + Box 4
- + CAAT-box
- + CCGTCC motif
- + CCGTCC-box
- + ERE
- + G-Box
- + G-box
- + GT1-motif
- + Gap-box
- + I-box
- + LTR
- + MRE
- + MYB
- + MYB-like sequence
- + MYC
- + Myb
- + STRE
- + Sp1
- + TATA
- + TATA-box
- + TC-rich repeats
- + TCA
- + TCT-motif
- + TGA-element
- + Unnamed__1
- + Unnamed__4
- + Unnamed__6
- + W box
- + WRE3
- + WUN-motif
- + chs-CMA1a
- + chs-CMA2c
- + circadian

Motifs Found

- +
- + ABRE
- + ABRE3a
- + ABRE4
- + AE-box
- + ARE
- + AT~TATA-box
- + AuxRR-core
- + Box 4
- + CAAT-box
- + CGTCA-motif
- + DRE core
- + ERE
- + G-Box
- + G-box
- + GARE-motif
- + GT1-motif
- + MBS
- + MYB
- + MYB-like sequence
- + MYC
- + Myb
- + Myb-binding site
- + Myc
- + STRE
- + TATA
- + TATA-box
- + TC-rich repeats
- + TCA
- + TCA-element
- + TCT-motif
- + TGA-element
- + TGACG-motif
- + Unnamed__1
- + Unnamed__4
- + Unnamed__6
- + W box
- + WUN-motif
- + as-1
- + circadian

Motifs Found

- +
- + A-box
- + AAGAA-motif
- + ABRE
- + AE-box
- + ARE
- + AT-rich element
- + ATCT-motif
- + AT~TATA-box
- + Box 4
- + Box II
- + CAAT-box
- + CCAAT-box
- + CCGTCC motif
- + CCGTCC-box
- + CGTCA-motif
- + ERE
- + G-Box
- + GATA-motif
- + GCN4_motif
- + GT1-motif
- + MBS
- + MYB
- + MYB recognition site
- + MYB-like sequence
- + MYC
- + Myb
- + O2-site
- + OCT
- + P-box
- + STRE
- + TATA
- + TATA-box
- + TC-rich repeats
- + TCA-element
- + TCT-motif
- + TGA-element
- + TGACG-motif
- + Unnamed__1
- + Unnamed__2
- + Unnamed__4
- + W box

+ WRE3
+ WUN-motif
+ as-1

Motifs Found

+
+ AAGAA-motif
+ ABRE
+ ABRE3a
+ ABRE4
+ AE-box
+ ARE
+ AT~TATA-box
+ AuxRR-core
+ Box 4
+ CAAT-box
+ CGTCA-motif
+ DRE core
+ DRE1
+ ERE
+ G-box
+ GATA-motif
+ GT1-motif
+ MBS
+ MYB
+ MYB-like sequence
+ MYC
+ Myb
+ P-box
+ STRE
+ TATA-box
+ TC-rich repeats
+ TCA-element
+ TCCC-motif
+ TCT-motif
+ TGA-element
+ TGACG-motif
+ Unnamed__4
+ W box
+ WUN-motif
+ as-1
+ circadian

Motifs Found

- +
- + AAGAA-motif
- + ABRE
- + ABRE3a
- + ABRE4
- + ACE
- + AE-box
- + ARE
- + AT-rich element
- + AT~TATA-box
- + Box 4
- + CAAT-box
- + CGTCA-motif
- + ERE
- + G-Box
- + G-box
- + GATA-motif
- + GT1-motif
- + MRE
- + MYB
- + MYC
- + STRE
- + TATA
- + TATA-box
- + TC-rich repeats
- + TCA-element
- + TCCC-motif
- + TCT-motif
- + TGA-element
- + TGACG-motif
- + Unnamed__1
- + Unnamed__4
- + Unnamed__6
- + W box
- + WUN-motif
- + as-1
- + circadian

Motifs Found

+
+ AAGAA-motif
+ ABRE
+ AC-I
+ AE-box
+ ARE
+ AT1-motif
+ AT~TATA-box
+ CAAT-box
+ CCAAT-box
+ CGTCA-motif
+ G-Box
+ GATA-motif
+ GT1-motif
+ HD-Zip 1
+ I-box
+ LAMP-element
+ LTR
+ MBS
+ MYB
+ MYB recognition site
+ MYB-like sequence
+ MYC
+ Myb
+ Myb-binding site
+ STRE
+ TATA
+ TATA-box
+ TCA-element
+ TCT-motif
+ TGA-element
+ TGACG-motif
+ Unnamed__1
+ Unnamed__4
+ Unnamed__6
+ W box
+ as-1

Motifs Found

- +
- + A-box
- + ABRE
- + ARE
- + AT~TATA-box
- + AuxRR-core
- + Box 4
- + CAAT-box
- + CAT-box
- + CCGTCC motif
- + CCGTCC-box
- + CGTCA-motif
- + ERE
- + G-Box
- + G-box
- + GATA-motif
- + GCN4_motif
- + GT1-motif
- + HD-Zip 3
- + LAMP-element
- + MBS
- + MYB
- + MYB-like sequence
- + MYC
- + Myb
- + Myb-binding site
- + O2-site
- + P-box
- + TATA-box
- + TC-rich repeats
- + TCA
- + TCT-motif
- + TGA-element
- + TGACG-motif
- + Unnamed__1
- + Unnamed__2
- + Unnamed__4
- + W box
- + WRE3
- + as-1
- + chs-CMA1a

Motifs Found

+

+ 3-AF3 binding site

+ AAGAA-motif

+ ABRE

+ ABRE3a

+ ABRE4

+ AC-II

+ AP-1

+ ARE

+ AT-rich element

+ AT~TATA-box

+ Box 4

+ CAAT-box

+ CGTCA-motif

+ CTAG-motif

+ ERE

+ G-Box

+ G-box

+ GARE-motif

+ GT1-motif

+ LAMP-element

+ MRE

+ MYB

+ MYB-like sequence

+ MYC

+ Myb

+ Myb-binding site

+ Myc

+ O2-site

+ TATA

+ TATA-box

+ TCA-element

+ TGA-element

+ TGACG-motif

+ Unnamed__1

+ Unnamed__4

+ Unnamed__6

+ W box

+ WRE3

+ WUN-motif

+ as-1

Motifs Found

- +
- + 3-AF3 binding site
- + A-box
- + AAGAA-motif
- + ABRE
- + ABRE3a
- + ABRE4
- + AE-box
- + ARE
- + ATCT-motif
- + AT~TATA-box
- + Box 4
- + CAAT-box
- + CCAAT-box
- + CCGTCC motif
- + CCGTCC-box
- + CGTCA-motif
- + ERE
- + G-box
- + GA-motif
- + GARE-motif
- + GATA-motif
- + GCN4_motif
- + GT1-motif
- + I-box
- + LAMP-element
- + LTR
- + MYB
- + MYB recognition site
- + MYB-like sequence
- + MYC
- + Myb
- + Myb-binding site
- + Myc
- + RY-element
- + TATA
- + TATA-box
- + TCCC-motif
- + TCT-motif
- + TGACG-motif
- + Unnamed__1
- + Unnamed__10

+		Unnamed__12
+		Unnamed__14
+		Unnamed__4
+		Unnamed__8
+		W box
+		WUN-motif
+		as-1

Motifs Found

- +
- + AAGAA-motif
- + ABRE
- + AE-box
- + ARE
- + AT~TATA-box
- + AuxRR-core
- + Box 4
- + Box III
- + CAAT-box
- + CGTCA-motif
- + G-Box
- + G-box
- + GA-motif
- + GCN4_motif
- + GT1-motif
- + Gap-box
- + I-box
- + LTR
- + MBSI
- + MRE
- + MYB
- + MYB-like sequence
- + MYC
- + Myb
- + Myb-binding site
- + Myc
- + TATA
- + TATA-box
- + TC-rich repeats
- + TCA
- + TGACG-motif
- + Unnamed__1
- + Unnamed__4
- + WRE3
- + as-1

Motifs Found

- +
- + AAGAA-motif
- + ABRE
- + ABRE3a
- + ABRE4
- + ARE
- + AT~TATA-box
- + Box 4
- + CAAT-box
- + CAT-box
- + CGTCA-motif
- + CTAG-motif
- + ERE
- + G-Box
- + G-box
- + GATA-motif
- + GT1-motif
- + LAMP-element
- + LTR
- + MBS
- + MYB
- + MYB-like sequence
- + MYC
- + Myb
- + Myb-binding site
- + Myc
- + TATA
- + TATA-box
- + TC-rich repeats
- + TCA
- + TCT-motif
- + TGA-element
- + TGACG-motif
- + Unnamed__1
- + Unnamed__10
- + Unnamed__12
- + Unnamed__14
- + Unnamed__4
- + Unnamed__8
- + WUN-motif
- + as-1
- + chs-CMA1a

+ circadian
