# Supplementary material for: Characterization of the TCP Gene Family in Chrysanthemum nankingense and the Role of CnTCP4 in Cold Tolerance
Source: Plants (Basel). 2022 Mar 30;11(7):936. doi: 10.3390/plants11070936 (PMC9002959; doi:10.3390/plants11070936)
Supplement: Supplementary file 1 [file plants-11-00936-s001.zip › Tian_chang_Supplementary Materials_legend.pdf]

Supplementary Materials:

Figure S1. Evolutionary tree of TCP proteins. The phylogenetic tree was generated with the MEGA7.0 program using the bHLH amino acid sequences from *Helianthus annuus* (Ha), *Chrysanthemum nankingense*, and *Arabidopsis thaliana*, with 1000 bootstrap replicates.

Figure S2. Gene structure of TCPs. UTRs and CDS are indicated by the green and yellow boxes, respectively. Introns are indicated by the thin gray lines.

Figure S3. Co-expression network analysis of TCP proteins and correlated proteins. A, TCPs co-expressed with correlated proteins. B, TCPs co-expressed with each other.

Figure S4. Expression of cold-related genes after cold stress and identified of *OECnTCP4*. A, RT-PCR of *CnTCP4* in wild-type and *OECnTCP4* Arabidopsis plants. B, 8-day-old seedlings of *OECnTCP4 Arabidopsis thaliana* were exposed to 4°C for 0, 6, and 24 h. Expression of cold-related genes *AtCOR15* and *AtKIN1* were assessed with quantitative real-time RT-PCR. *ACTIN8* was used as the internal control. Values are means ± SD.

Figure S5. Overexpression of *CnTCP4* and *CnTCP13* delayed bolting in *Arabidopsis thaliana*. Values are means ± SD. The different letters represent significant difference at $p < 0.01$, according to the paired-samples *t*-test.

Table S1. Primers and sequences used in this research.

Table S2. Distance and homology matrix of 171 sequences were generated with the DNAMAN program using the full-length amino acid sequences from *Glycine max*, *Oryza sativa*, *Zea mays*, *Chrysanthemum nankingense*, and *Arabidopsis thaliana*.

Table S3. The distance and homology matrix of 171 sequences were generated with the DNAMAN program using the bHLH amino acid sequences from *Glycine max*, *Oryza sativa*, *Zea mays*, *Chrysanthemum nankingense*, and *Arabidopsis thaliana*.

Table S4. Distance and homology matrix of 23 sequences were generated with the DNAMAN program using the full-length and bHLH amino acid sequences from *Chrysanthemum nankingense*.

Table S5. Binding site-prediction results of *TCP* promoters from *Chrysanthemum nankingense* and *Arabidopsis thaliana* using the PlantRegMap website

Table S6. *Cis*-acting regulatory element-prediction results of *TCP* promoters from *Chrysanthemum nankingense* and *Arabidopsis thaliana* using the PlantCARE website
