# Supplementary figures and images for: Characterization of the TCP Gene Family in Chrysanthemum nankingense and the Role of CnTCP4 in Cold Tolerance
Source: Plants (Basel). 2022 Mar 30;11(7):936. doi: 10.3390/plants11070936 (PMC9002959; doi:10.3390/plants11070936)

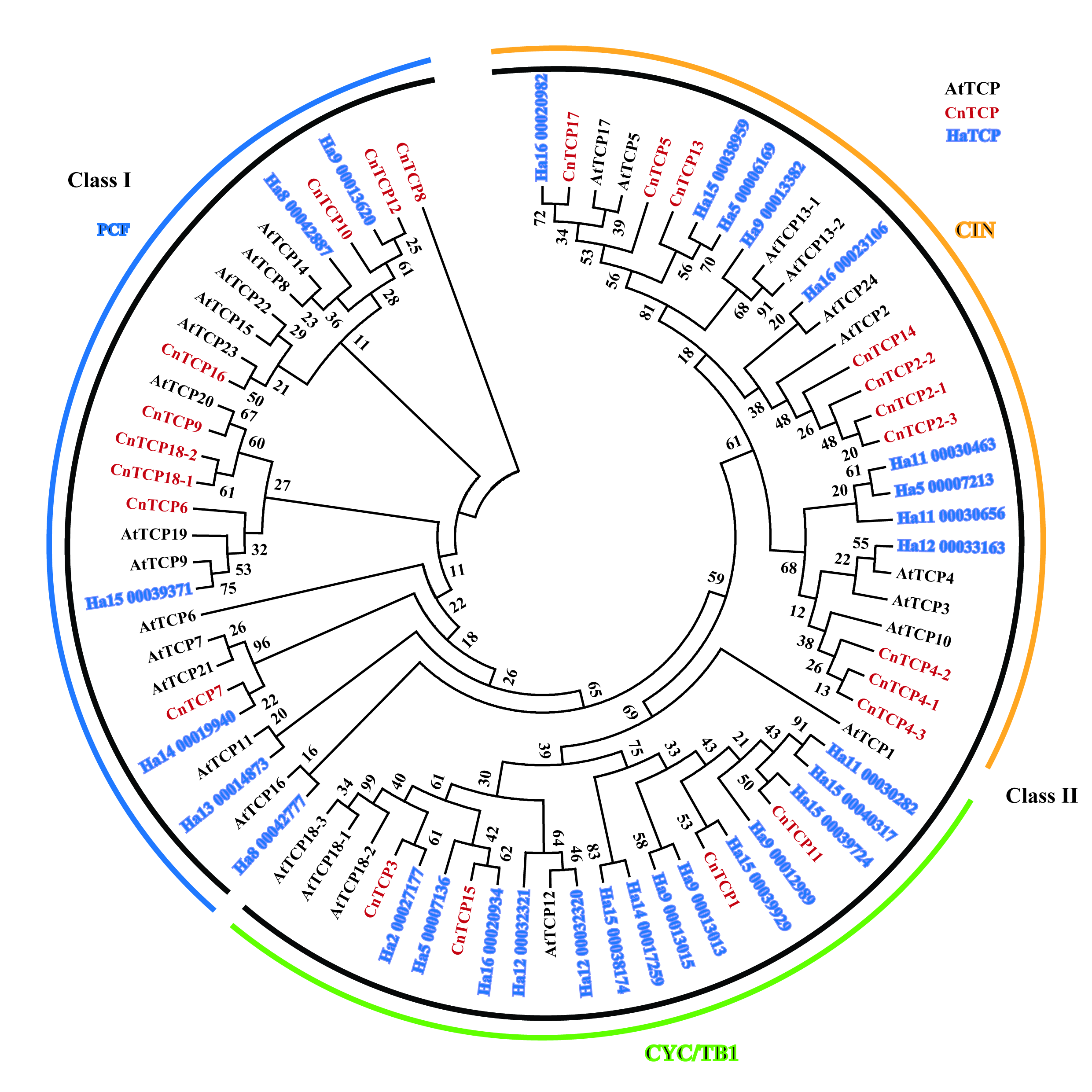

Supplement: Supplementary file 1 [file plants-11-00936-s001.zip › Figure S1.tif]

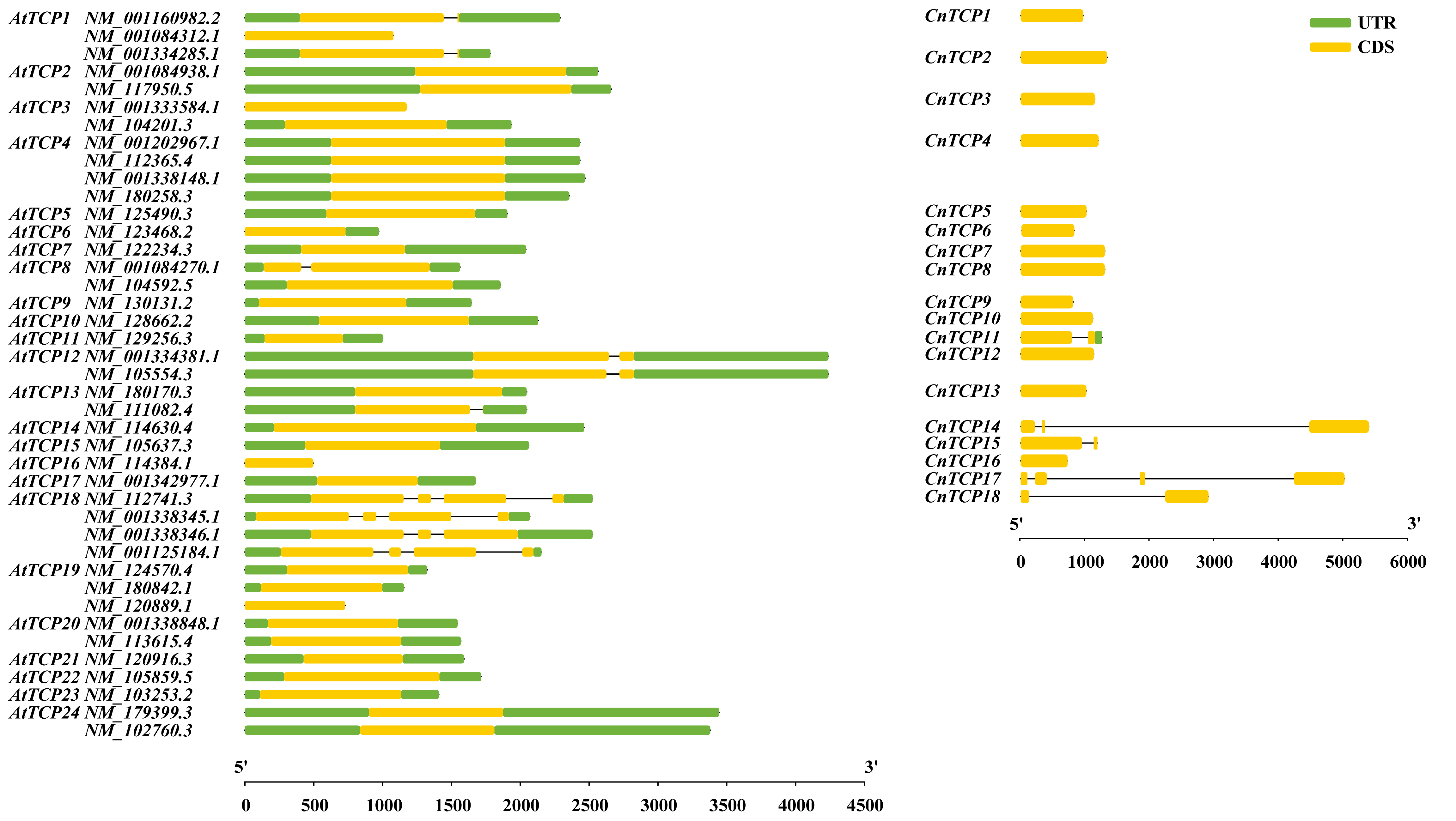

Supplement: Supplementary file 1 [file plants-11-00936-s001.zip › Figure S2.tif]

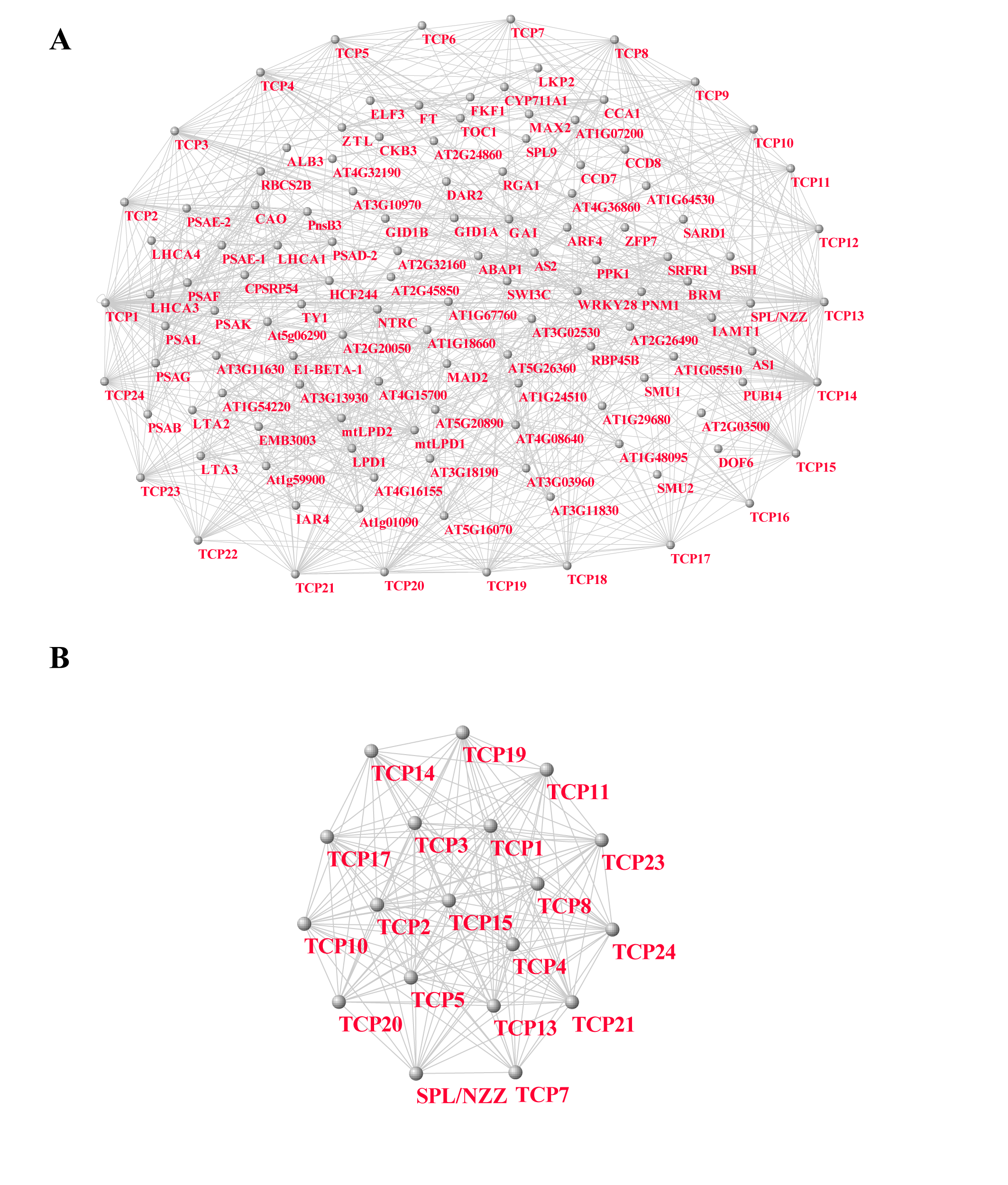

Supplement: Supplementary file 1 [file plants-11-00936-s001.zip › Figure S3.tif]

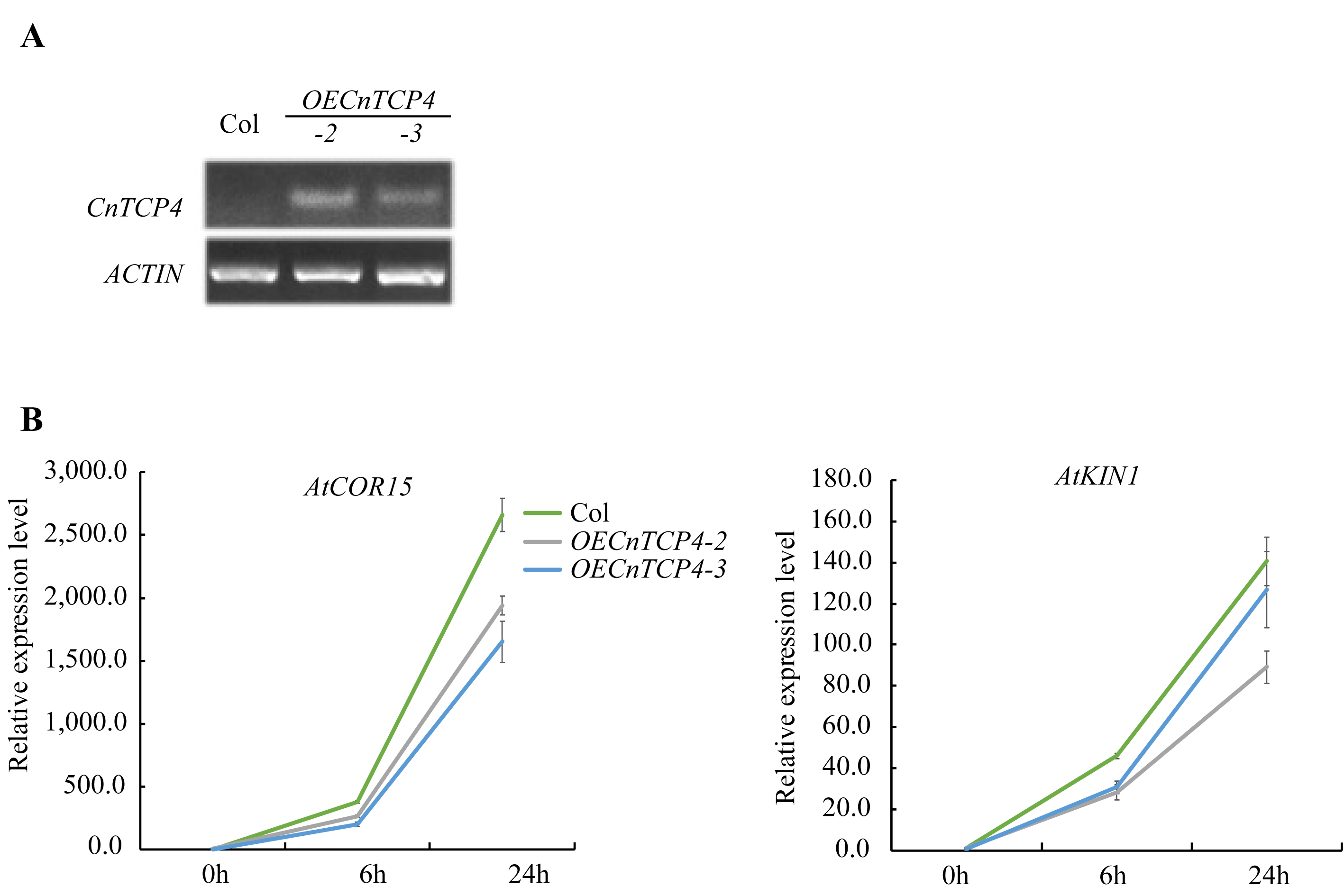

Supplement: Supplementary file 1 [file plants-11-00936-s001.zip › Figure S4.tif]

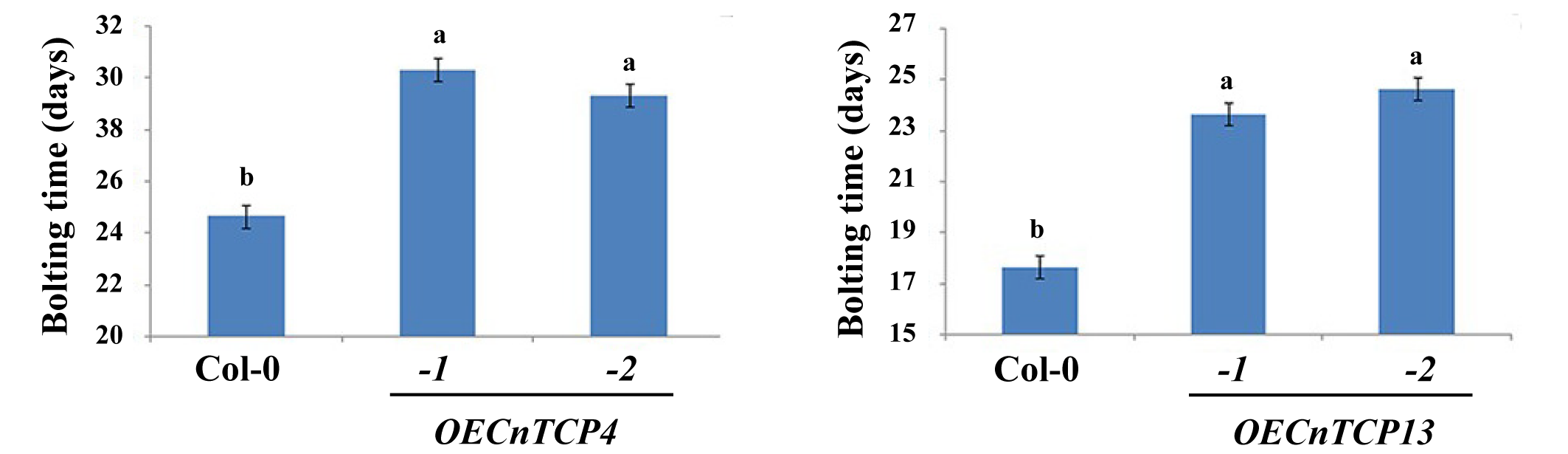

Supplement: Supplementary file 1 [file plants-11-00936-s001.zip › Figure S5.tif]
